# Life Course Trajectories of Cardiovascular Risk Factors in Women With and Without Hypertensive Disorders in First Pregnancy: The HUNT Study in Norway

**DOI:** 10.1161/JAHA.118.009250

**Published:** 2018-07-27

**Authors:** Eirin B. Haug, Julie Horn, Amanda R. Markovitz, Abigail Fraser, Lars J. Vatten, Corrie Macdonald‐Wallis, Kate Tilling, Pål R. Romundstad, Janet W. Rich‐Edwards, Bjørn O. Åsvold

**Affiliations:** ^1^ Department of Public Health and Nursing NTNU Norwegian University of Science and Technology Trondheim Norway; ^2^ Department of Obstetrics and Gynecology Levanger Hospital Nord‐Trøndelag Hospital Trust Levanger Norway; ^3^ Harvard T.H. Chan School of Public Health Harvard University Boston MA; ^4^ Division of Women's Health Brigham and Women's Hospital and Harvard Medical School Boston MA; ^5^ MRC Integrative Epidemiology Unit and Population Health Sciences Bristol Medical School University of Bristol United Kingdom; ^6^ Department of Endocrinology St. Olavs Hospital Trondheim University Hospital Trondheim Norway

**Keywords:** cardiovascular risk factors, epidemiology, hypertensive disorders of pregnancy, life course, Cardiovascular Disease, Epidemiology, Pregnancy, Women

## Abstract

**Background:**

Women with hypertensive pregnancy disorders have adverse levels of cardiovascular risk factors. It is unclear how this adverse risk factor profile evolves during adult life. We compared life course trajectories of cardiovascular risk factors in women with preeclampsia or gestational hypertension in their first pregnancy to normotensive women.

**Methods and Results:**

We linked information on cardiovascular risk factors from the population‐based HUNT (Nord‐Trøndelag Health Study) surveys with pregnancy information from the Medical Birth Registry of Norway. Trajectories of cardiovascular risk factors were constructed for 22 308 women with a normotensive first pregnancy; 1092 with preeclampsia, and 478 with gestational hypertension in first pregnancy. Already before first pregnancy, women with preeclampsia in their first pregnancy had higher measures of adiposity, blood pressure, heart rate, and serum lipids and glucose compared with women with a normotensive first pregnancy. After first pregnancy, there was a parallel development in cardiovascular risk factor levels, but women with a normotensive first pregnancy had a time lag of >10 years compared with the preeclampsia group. There were no clear differences in risk factor trajectories between women with gestational hypertension and women with preeclampsia.

**Conclusions:**

Women with hypertensive pregnancy disorders in their first pregnancy had an adverse cardiovascular risk factor profile before pregnancy compared with normotensive women, and the differences persisted beyond 50 years of age. Hypertensive disorders in pregnancy signal long‐term increases in modifiable cardiovascular risk factors, and may be used to identify women who would benefit from early prevention strategies.

**Clinical Perspective**

**What Is New?**

- In women with hypertensive disorders of pregnancy (HDP), adverse levels of adiposity, blood pressure, heart rate, serum lipids and glucose were present before first pregnancy and remained higher compared with other women beyond 50 years of age.
- Progression of cardiovascular risk factors throughout the age interval 20 to 60 years occurred mostly in parallel for women with and without a history of HDP, with greater increases in systolic blood pressure and adiposity in women with a history of HDP.

**What Are the Clinical Implications?**

- Women with a history of HDP may be expected to pass beyond treatment thresholds of cardiovascular risk factors at least 10 years earlier than women with normotensive pregnancy.
- Our results suggest that women with a history of HDP may benefit from early screening and intervention programs that seek to lower the levels of cardiovascular risk factors.

## Introduction

Cardiovascular disease (CVD) accounts for ≈1 in 3 deaths in women.^1^ Hypertensive disorders of pregnancy (HDP), including preeclampsia and gestational hypertension, occur in up to 10% of all pregnancies.^2^ Pregnancy may serve as a stress test of maternal cardiovascular health, where HDP may indicate a reduced ability to accommodate the extra cardiovascular and metabolic challenges of pregnancy.^3^ HDP may reveal a phenotype predisposed to CVD, and may therefore be used to identify women who would benefit from early screening and preventive efforts. A history of HDP has been included as a cardiovascular risk factor in CVD prevention guidelines in the United States since 2011^4^ and in Europe since 2016.^5^ Yet there is little evidence and no consensus on how to tailor CVD screening and prevention in women with a history of HDP. Although previous studies reported adverse cardiovascular risk factor profiles in women with HDP both before and after pregnancy,^6^, ^7^, ^8^, ^9^, ^10^, ^11^, ^12^, ^13^, ^14^, ^15^, ^16^, ^17^ detailed knowledge on how different cardiovascular risk factors develop throughout life is lacking. In particular, it is unclear when in life the elevated cardiovascular risk profile manifests itself in women with a history of HDP, and whether and how this profile may change from before to after a pregnancy complicated with HDP, and also how differences in cardiovascular risk factors between women with and without HDP may evolve postpartum.

To our knowledge, no longitudinal studies have examined long‐term trajectories of cardiovascular risk factors among women with a history of HDP from before first pregnancy until middle age. In the HUNT (Nord‐Trøndelag Health Study) cohort in Norway, we recently observed that higher blood pressure in women with a history of HDP manifests before first pregnancy and lasts beyond 60 years of age.^18^ In the present study, we examine the life course trajectories from before first pregnancy and until 60 years of age for a broad range of cardiovascular risk factors in women with and without HDP in their first pregnancy.

## Methods

Data from the HUNT Study used in research projects will when reasonably requested by others be made available upon request to the HUNT Data Access Committee (hunt@medisin.ntnu.no). The HUNT data access information (available here: http://www.ntnu.edu/hunt/data) describes in detail the policy regarding data availability.

All procedures performed in studies involving human participants were in accordance with the ethical standards of the institutional and/or national research committee and with the 1964 Helsinki declaration and its later amendments or comparable ethical standards. Informed consent was obtained from all individual participants included in the study.

### Study Population

**Figure 1:**
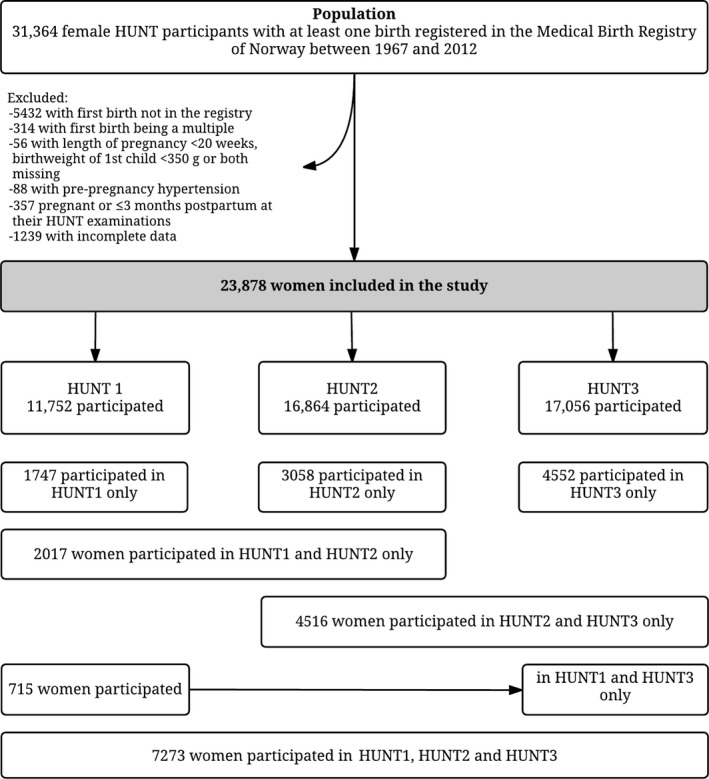
Flow chart of study population. HUNT indicates Nord‐Trøndelag Health Study.

The HUNT study is a longitudinal population study that has invited all adult inhabitants 20 years and older in Nord‐Trøndelag county, Norway, to take part in health surveys since the 1980s. The surveys include questionnaires, interviews, blood sampling, and clinical measurements.^19^, ^20^, ^21^ So far, 3 HUNT surveys have been conducted: HUNT1 1984–1986,^20^ HUNT2 1995–1997,^21^ and HUNT3 2006–2008.^19^ The predominantly (>97% at the time of HUNT2) white population in Nord‐Trøndelag is considered to be fairly representative for Norway as a whole.^21^ The Medical Birth Registry of Norway (MBRN) has recorded all births in the country since 1967 and provides detailed information on maternal and child characteristics.^22^ Information from the MBRN and HUNT was linked using the 11‐digit unique personal identification number that is allocated to all Norwegian citizens. In total, 25 932 women whose first delivery had been recorded in the MBRN between its inception in 1967 and 2012 had also taken part in at least 1 HUNT survey between 1984 and 2008. Among them, we excluded 314 women whose first birth was a multiple and, since preeclampsia and gestational hypertension cannot be diagnosed before 20 weeks of gestation, we further excluded 56 women with either gestational length <20 weeks, offspring birth weight <350 g, or missing information on both gestational length and offspring birth weight. In addition, we excluded 88 women who had a pre‐first pregnancy diagnosis of hypertension and 357 women who were pregnant or <3 months postpartum at all their HUNT examinations. Lastly, we excluded 1239 women because of incomplete information on smoking or education or because they had no cardiovascular risk factor measurements, leaving 23 878 women for statistical analysis (Figure 1).

### Exposures and Covariates

Diagnoses of preeclampsia and gestational hypertension in first pregnancy were retrieved from the MBRN, which uses internationally recommended diagnostic criteria^2^: Gestational hypertension was generally defined as de novo hypertension (≥140 mm Hg systolic and/or ≥90 mm Hg diastolic) after 20 weeks of gestation, and preeclampsia also required proteinuria (300 mg/24 h or ≥1+ on the dipstick test). Validation studies^23^, ^24^ within the HUNT study population have estimated the positive predictive values of the preeclampsia and gestational hypertension diagnoses in the MBRN to be 88% and 68%, respectively.

From the HUNT questionnaires and interviews, we retrieved self‐reported information on use of antihypertensive medication, diabetes mellitus, ever daily smoking, hours since last meal, highest obtained educational level, and work titles. Since education level was not available in HUNT3, we derived educational level from work titles based on recommendations from Statistics Norway^25^ for 5546 women.

### Cardiovascular Risk Factors

Blood sampling and clinical measurements were performed by trained staff at the HUNT examination stations. Height and weight were measured with the person wearing light clothes and no shoes and were rounded to the nearest cm (height) and half kilo (weight). Body mass index (BMI) was calculated as weight (in kg) divided by the squared value of height (in m), and obesity was defined as BMI ≥30 kg/m^2^. For 12 832 women in HUNT3, we also calculated BMI at age 18 years using self‐reported height and weight at age 18 years. Blood pressure in HUNT1 was measured manually 2 times at 1‐minute intervals using a sphygmomanometer after the person had come to rest, and we used the mean value of these 2 measurements in our analysis. In HUNT2 and HUNT3, blood pressure was measured 3 times at 1‐minute intervals using an automatic oscillometric method (Dinamap, Critikon, FL) after the person had come to rest, with cuff size adjusted to arm circumference. We used the mean of the second and third measurement, except for 2135 women in HUNT3 who lacked the third measurement because of sick leave among staff; for them, we used the second measurement only. Based on recommendations by Cui et al^26^ and Tobin et al,^27^ we added 10 mm Hg to systolic and 5 mm Hg to diastolic blood pressure levels for 2137 women who reported taking antihypertensive medication. We classified women as having hypertension if they reported taking antihypertensive medication, or whose blood pressure was either ≥140 mm Hg systolic or ≥90 mm Hg diastolic. Resting heart rate in beats/min was measured 1 time in HUNT1 and 3 times in HUNT2 and HUNT3 using the same devices as for blood pressure described above. For HUNT2 and HUNT3, we used the mean of the second and third measurements. Waist and hip circumference (available in HUNT2 and HUNT3) were measured to the nearest centimeter while the person was standing with arms hanging down at the height of the umbilicus (waist circumference) or at the thickest part of the hip (hip circumference).

All serum analyses were performed in nonfasting samples at the Central Laboratory, Levanger Hospital, Nord‐Trøndelag Hospital Trust using a Hitachi 911 Autoanalyzer in HUNT2 and Architect cSystems ci8200 in HUNT3. All analyses were performed in fresh serum samples, except C‐reactive protein (CRP) in HUNT2, which was measured after 2 years of serum storage at −80°C. Serum total and high‐density lipoprotein (HDL) cholesterol and triglycerides were analyzed using enzymatic colorimetric methods (Boeheringer Mannheim, Germany) in HUNT2. In HUNT3, HDL cholesterol was measured with an accelerator selective detergent methodology, total cholesterol was analyzed by a cholesterol esterase methodology, and triglycerides were measured by a glycerol phosphate oxidase methodology, all by equipment from Abbott, Clinical Chemistry, USA. Non‐HDL cholesterol was calculated as the difference between total and HDL cholesterol. High‐sensitive CRP was measured in participants from 4 out of 24 municipalities (n=2766) in HUNT2 using a CRP ultrasensitive assay (Tina‐quant(R); Roche, Basel, Switzerland). In HUNT3, CRP was measured in everyone using a latex immunoassay (Abbott, Clinical Chemistry, USA). In HUNT2 and HUNT3, serum glucose was measured for all persons using an enzymatic hexokinase method. In HUNT1, capillary glucose was measured at the examination stations in participants >40 years (Reflocheck‐Glucose; Boehringer Mannheim, Germany), and for the analysis of mean glucose levels, we transformed capillary levels to equate serum values (in mmol/L) by multiplying by 1.11.^28^ In HUNT1, fasting capillary glucose was measured in persons with capillary glucose ≥8.0 mmol/L at the initial examination, and a 2‐hour oral glucose tolerance test was given if fasting capillary glucose was <7.0 mmol/L. If capillary glucose concentrations indicated diabetes mellitus (≥7.0 mmol/L fasting or ≥11.1 mmol/L after 2 hours), the corresponding serum glucose concentrations were measured. We defined diabetes mellitus by self‐report (all HUNT surveys), nonfasting serum glucose ≥11.1 mmol/L (HUNT2 or HUNT3), or fasting serum glucose ≥7.0 mmol/L or 2‐hour postload serum glucose ≥11.1 mmol/L (HUNT1). Serum creatinine was measured with the Jaffe method in HUNT2 (Roche Diagnostics, Mannheim, Germany) and with an alkaline picrate methodology in HUNT3 (Abbott, Clinical Chemistry, USA), and calibrated to isotope‐dilution mass‐spectroscopy level using an enzymatic method (Roche).^29^ Estimated glomerular filtration rate in mL/min per 1.73 m^2^ was calculated using the Chronic Kidney Disease Epidemiology consortium formula,^30^ which takes account of creatinine, age, and sex.

### Statistical Analysis

Life course trajectories of cardiovascular risk factors were modeled using linear spline mixed‐effects models,^31^ except for CRP, which had a limited number of repeated measurements and was modeled using a linear spline regression model with a cluster‐robust estimate of variance (Huber/White sandwich estimate). The linear spline mixed‐effects models included subject‐specific (random) intercepts and slopes to account for up to 3 repeated dependent observations per woman and facilitated estimation of within‐woman trajectories.^32^ Linear splines defined by age intervals were used in order to allow for nonlinear change in the cardiovascular risk factor over time. The most appropriate age intervals were determined for each cardiovascular risk factor by comparing performance of models with 2, 4, 5, 6, 8, and 10 years age intervals using the Bayesian Information Criterion. On the basis of this, 10‐year age intervals up to age 70 years were selected for all cardiovascular risk factors. All models adjusted for highest obtained education level (lower secondary [≤9 years], upper secondary [10–12 years], and tertiary [college or university]), ever daily smoking, HUNT survey, and age at first birth while also allowing the age‐dependent change in cardiovascular risk factor (linear spline) to vary by exposure status and by different levels of these potential confounders. Analyses of glucose and triglycerides were additionally adjusted for number of hours since last meal (<1, 1, 2, 3, 4, 5, or ≥6 hours). We included 1 term describing the immediate change in cardiovascular risk factor level from pre‐ to post‐first pregnancy, and another indicating the change in increase/decrease per year (slope) from pre‐ to post‐first pregnancy. We allowed both terms to vary by whether the woman's first pregnancy was complicated by preeclampsia/gestational hypertension or was normotensive and by different levels of education, smoking, and age at first birth. All women aged 20 to 82 years old were included in the analysis, but because of limited data for women >60 years, we show predicted cardiovascular risk factor trajectories for the age range 20 to 60 years (18–60 for BMI, because of the available self‐reported height and weight at age 18 years). We had insufficient data to model the risk factor trajectories during pregnancy and placed gaps in the predicted trajectories corresponding to the first pregnancy and a 3 months postpartum period. We predicted the risk factor trajectories as if the woman had her first birth at age 23 years, the median age at first birth in our study population, and with all the remaining covariates set at their sample means. As a sensitivity analysis, we also modeled the same cardiovascular risk factor trajectories among women who had taken part in 2 or more HUNT surveys in order to examine the potential impact of including women with single measurements. In a separate analysis using logistic regression with cluster‐robust variance, we also estimated the probability of being obese, having hypertension or diabetes mellitus as a function of age adjusting for highest obtained education level, ever daily smoking, age at first birth, and HUNT survey. In an additional analysis among women with at least 2 pregnancies, we examined whether repeat preeclampsia was associated with a more adverse cardiovascular risk profile. In this analysis, we contrasted cardiovascular risk trajectories in women having preeclampsia in both first and second pregnancy with women having preeclampsia in one of these pregnancies. Since the risk of preeclampsia is associated with pregnancy interval,^33^, ^34^ we additionally adjusted for time between the first and second pregnancy in this analysis. All analyses were performed using Stata IC 14 and MLwiN version 2.34^35^ via the runmlwin^36^ command in Stata.

## Results

Characteristics of our study population are given in the Table. Among 23 878 women, 1092 (5%) had preeclampsia and 478 (2%) had gestational hypertension in their first pregnancy. Cardiovascular risk factors were measured within a time span of 20 years before to 40 years after first birth. In total, 7273 (30%) women participated in all 3 HUNT surveys, 7248 (30%) took part in 2 and 9357 (39%) only participated in 1 HUNT survey. Median age at first birth was similar for women with preeclampsia, gestational hypertension, and normotension in first pregnancy. Preterm delivery and small for gestational age offspring were more common in preeclamptic pregnancies. The numbers of women and measurements included in each of the cardiovascular risk factor analyses are given in Table S1, and Figure S1 displays the distribution of observations by participation age and HUNT survey.

**Table 1:** Descriptive Characteristics of the Study Population

	Hypertension Status of First Pregnancy		
	Normotension (n=22 308)	Gestational Hypertension (n=478)	Preeclampsia (n=1092)
Maternal characteristics			
Birth year, median (IQR)	1959 (1951–1968)	1957 (1951–1966)	1962 (1953–1970)
Age at first birth, median (IQR)	23 (20–26)	24 (21–27)	24 (21–27)
Ever daily smoking, n (%)			
No	9132 (41)	240 (50)	585 (54)
Yes	13 176 (59)	238 (50)	507 (46)
Education, n (%)			
Lower secondary (≤9 y)	3737 (17)	89 (19)	177 (16)
Upper secondary (10–12 y)	10 540 (47)	217 (45)	551 (50)
Tertiary (>12 y)	8031 (36)	172 (36)	364 (33)
Ever use of antihypertensive medication, n (%)			
No	20 271 (91)	332 (69)	775 (71)
Yes	2033 (9)	146 (31)	317 (29)
Missing	4 (0)	0 (0)	0 (0)
Age at first HUNT exam, median (IQR)	31 (26–37)	31 (26–37)	31 (26–36)
No. of HUNT exams, n (%)			
1	8701 (39)	177 (37)	479 (44)
2	6799 (30)	125 (26)	324 (30)
3	6808 (31)	176 (37)	289 (26)
HUNT exams relative to first pregnancy, n (%)			
Before first pregnancy only	1927 (9)	50 (10)	113 (10)
After first pregnancy only	18 166 (81)	380 (79)	847 (78)
Before and after first pregnancy	2215 (10)	48 (10)	132 (12)
First pregnancy characteristics			
Gestational length in wks, n (%)			
<34	407 (2)	4 (1)	57 (5)
34–36	753 (3)	11 (2)	106 (10)
≥37	20 033 (90)	439 (92)	857 (78)
Missing	1115 (5)	24 (5)	72 (7)
Birth weight, n (%)^a^			
Small for gestational age	658 (3)	23 (5)	118 (11)
Normal	19 952 (89)	424 (89)	876 (80)
Large for gestational age	399 (2)	5 (1)	19 (2)
Missing	1299 (6)	26 (5)	79 (7)
Stillbirths, n (%)	193 (1)	2 (0)	21 (2)

IQR indicates interquartile range; HUNT, Nord‐Trøndelag Health Study.

a Small and large for gestational age were defined as >2 standard deviations away from the established mean birth weights by gestational age in the Medical Birth Registry of Norway.^37^

For the sake of clarity and brevity, we focus the description of the results on risk factor trajectories in women with preeclampsia compared with normotension in first pregnancy. However, throughout the analyses, results for women with gestational hypertension in first pregnancy were comparable to those for women with preeclampsia; full results for gestational hypertension are given in Figures S2 and S3. Where no reference to the order of the pregnancy is made, it is implied that we mean the first pregnancy.

**Figure 2:**
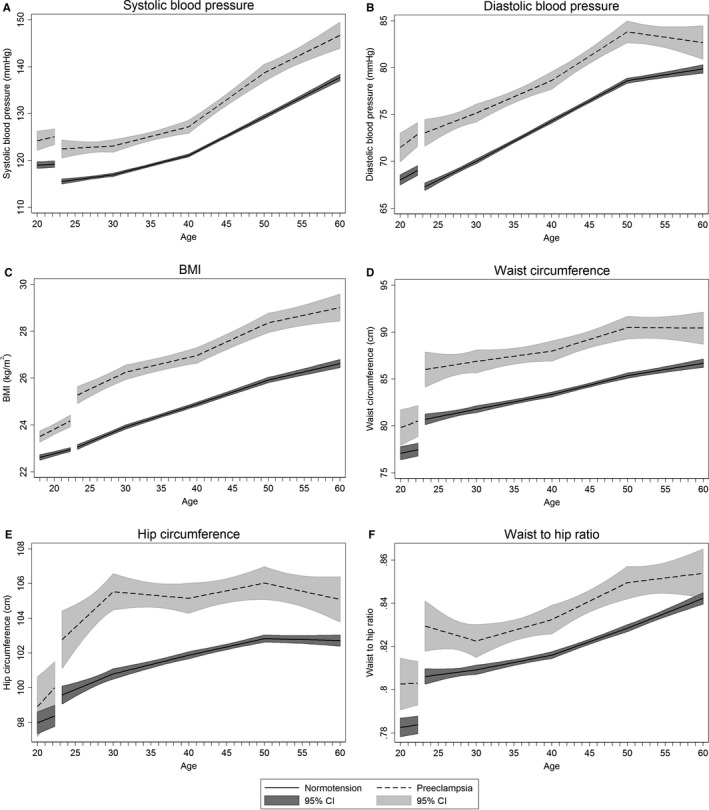
Life course trajectories of mean systolic blood pressure (A), diastolic blood pressure (B), BMI (C), waist circumference (D), hip circumference (E), and waist‐to‐hip ratio (F) for women with normotensive and preeclamptic first pregnancies. Estimates are adjusted for age at measurement, HUNT survey, highest obtained education level, age at first birth, and ever daily smoking. Covariates are fixed at their means with gaps in the graphs corresponding to the woman's first pregnancy, birth at age 23 years, and a 3‐month postpartum period. BMI indicates body mass index; CI, confidence interval; HUNT, Nord‐Trøndelag Health Study.

**Figure 3:**
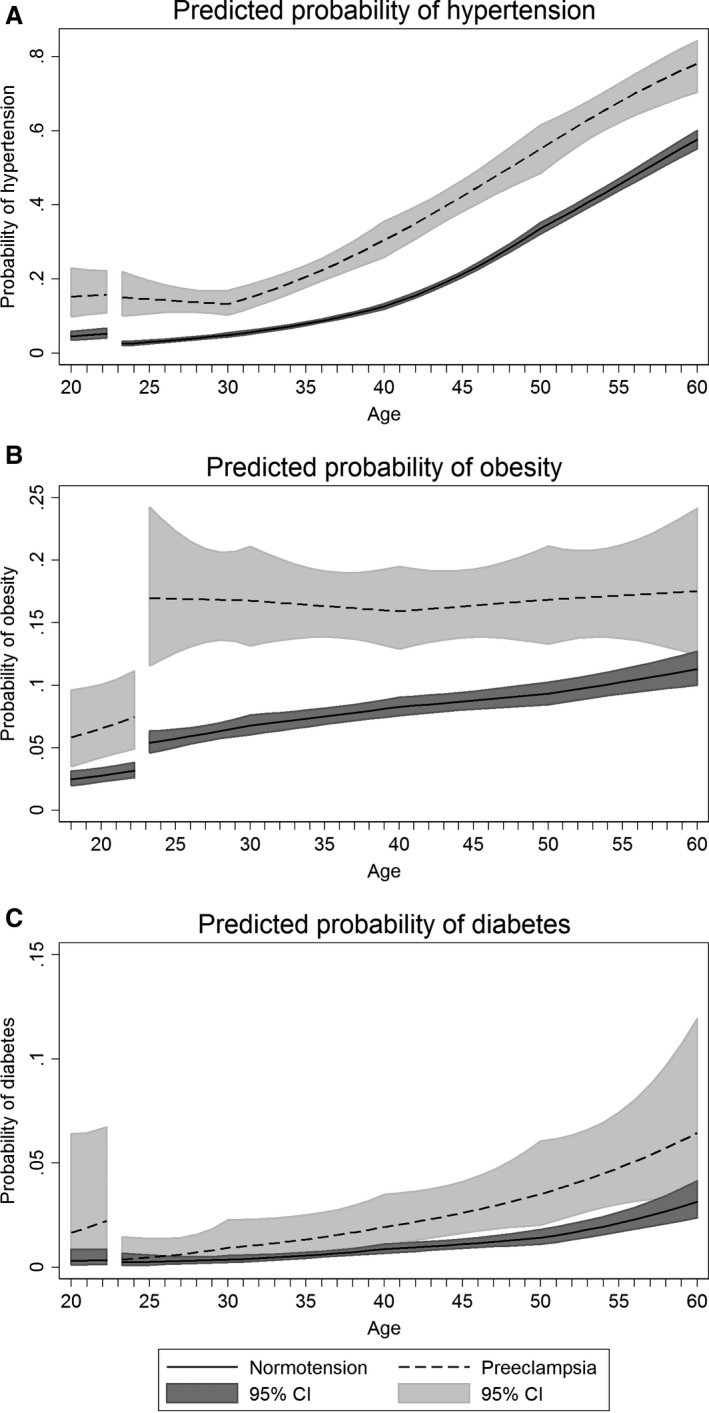
Population average predicted probabilities of hypertension (defined as current antihypertensive medication and/or blood pressure ≥140 mm Hg systolic or ≥90 mm Hg diastolic) (A), obesity (defined as a BMI ≥30 kg/m^2^) (B), and diabetes mellitus (defined as self‐reported diabetes mellitus, nonfasting serum glucose ≥11.1 mmol/L, fasting serum glucose ≥7.0 mmol/L, and/or 2‐hour postload serum glucose ≥11.1 mmol/L) (C) by age in women with normotensive and preeclamptic first pregnancies. Estimates are adjusted for age at measurement, HUNT survey, highest obtained education level, age at first birth, and ever daily smoking. Covariates are fixed at their means with gaps in the graphs corresponding to the woman's first pregnancy, birth at age 23, and a 3‐month postpartum period. BMI indicates body mass index; CI, confidence interval; HUNT, Nord‐Trøndelag Health Study.

At the age of 20 years, women who later had a preeclamptic pregnancy had 5.2 mm Hg (95% confidence interval [CI], 3.2–7.2) higher systolic and 3.5 mm Hg (95% CI, 2.0–5.0) higher diastolic blood pressure compared with women who later had a normotensive pregnancy (Figure 2A and 2B, Table S2). From pre‐ to postpregnancy, systolic blood pressure decreased both in women with preeclampsia and normotensive pregnancies, whereas diastolic blood pressure decreased only in women with normotensive pregnancies (Table S3). In the years following pregnancy, the increase in blood pressure was similar among women with preeclampsia and normotensive pregnancy, except that women with preeclampsia had a steeper increase in systolic blood pressure from 40 to 50 years of age (Table S4). By age 60 years, systolic blood pressure was 9.0 mm Hg (95% CI, 6.2–11.8) higher and diastolic blood pressure was 2.8 mm Hg (95% CI, 1.0–4.6) higher in women with preeclampsia compared with normotensive pregnancy (Table S2). The prevalence of hypertension was higher in women with preeclampsia compared with normotensive pregnancy throughout the entire age range, and the prevalence in women with preeclampsia increased more strongly after age 30 years, a decade earlier than the corresponding increase among women with normotensive pregnancy (Figure 3A, Table S5). At age 60 years, 78% (95% CI, 70–84) of women with a first preeclamptic pregnancy had hypertension, compared with 58% (95% CI, 55–60) of women with normotensive pregnancy (Figure 3A, Table S5).

BMI was 1.1 kg/m^2^ (95% CI, 0.8–1.3) higher at age 20 years in women with subsequent preeclampsia compared with women with a normotensive pregnancy (Figure 2C, Table S2). Up to pregnancy, and from pre‐ to immediately postpregnancy, BMI increased more steeply among women with preeclampsia (Tables S3 and S4). In the years after pregnancy, BMI increased linearly and in parallel in both groups, and at age 60 years, BMI was 2.4 kg/m^2^ (95% CI, 1.8–3.0) higher among women with preeclampsia compared to women with a normotensive pregnancy (Table S2). By age 60 years, the prevalence of obesity was 18% (95% CI, 12–24) in women with preeclampsia and 11% (95% CI, 10–13) in women with a normotensive pregnancy (Figure 3B and Table S5). Waist circumference and waist‐to‐hip ratio, measures of abdominal adiposity, were also consistently higher in women with a preeclampsia pregnancy, and increased with age in a broadly parallel fashion in both groups (Figure 2D through 2F, Tables S2 and S4).

**Figure 4:**
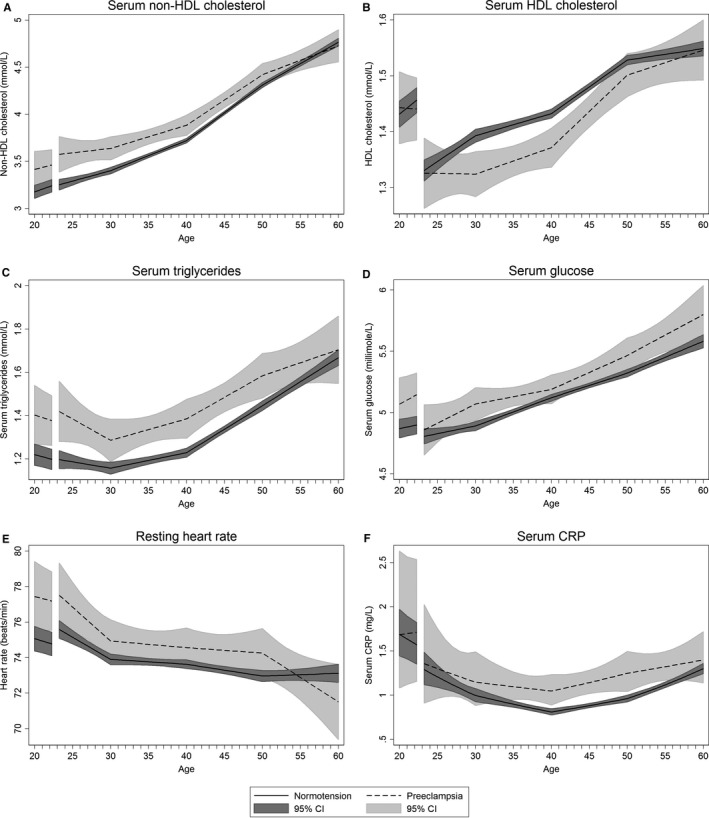
Life course trajectories of mean nonfasting serum non‐HDL (A) and HDL (B) cholesterol, triglycerides (C), and glucose (D), resting heart rate (E), and serum CRP (F) for women with normotensive and preeclamptic first pregnancies. Estimates are adjusted for age at measurement, HUNT survey highest obtained education level, age at first birth and ever daily smoking. Analyses of glucose and triglycerides were additionally adjusted for time since last meal. Covariates are fixed at their means with gaps in the graphs corresponding to the woman's first pregnancy, birth at age 23, and a 3‐month postpartum period. CRP is given as geometric mean. CI indicates confidence interval; CRP, C‐reactive protein; HDL, high‐density lipoprotein; HUNT, Nord‐Trøndelag Health Study.

Non‐HDL cholesterol was 0.24 mmol/L (95% CI, 0.05–0.43) higher at age 20 years among women with subsequent preeclampsia compared with a normotensive first pregnancy (Figure 4A, Table S2), and increased similarly in both groups until age 40 years (Figure 4A, Table S4). From 40 to 60 years, women with a normotensive pregnancy had a seemingly steeper rise, resulting in the 2 groups of women having similar non‐HDL cholesterol levels by age 60 years (Figure 4A, Table S2). HDL cholesterol levels were similar between the groups prepregnancy (Figure 4B, Table S2) and immediately postpregnancy. Women with preeclampsia then had lower HDL cholesterol until beyond 50 years of age compared with women with normotensive pregnancy (Figure 4B, Table S2). Triglyceride levels were 0.18 mmol/L (95% CI, 0.05–0.32) higher at age 20 in women who later had preeclampsia compared with normotensive pregnancy (Figure 4C, Table S2), and this difference between the groups remained broadly unchanged until 50 years of age. At age 60 years, the 2 groups of women had similar levels of all lipid subtypes (Figure 4A through 4C, Table S2).

Nonfasting serum glucose was ≈0.2 mmol/L higher in women with preeclampsia compared with normotensive pregnancies (Figure 4D, Table S2), and this difference was similar from ages 20 to 60 years. Diabetes mellitus prevalence rose faster in women with preeclampsia compared with normotensive pregnancy (Figure 3C). At age 60 years, 6% (95% CI, 3–12) of women with preeclampsia and 3% (95% CI, 2–4) of women with normotensive first pregnancies had diabetes mellitus (Table S5).

Resting heart rate was 2.4 beats/min (95% CI, 0.4–4.3) faster at age 20 in women with preeclampsia compared with normotensive pregnancy (Figure 4E, Table S2). After pregnancy, resting heart rate was 1 beat/min faster until 50 years of age in women with preeclampsia compared with normotensive pregnancy (Table S2). Prepregnancy CRP levels were similar in women with preeclampsia and normotensive pregnancy (Figure 4F, Table S2). Following pregnancy, CRP was higher in preeclamptic women, especially at age 30 to 55 years, but the CRP trajectories were less precise because of a lower number of measurements (Figure 4F, Table S2). Estimated glomerular filtration rate decreased in a linear fashion throughout the entire age‐interval in all women without any noticeable differences between women with normotension or preeclampsia in their first pregnancy (Figure S4).

For all the above‐described analyses except for CRP, we obtained similar results when restricting the analysis to women with 2 or more repeated measures (Figures S5 and S6).

The analysis of repeat exposure to preeclampsia included 121 women with preeclampsia in both first and second pregnancy, 929 women with preeclampsia in 1 of these pregnancies, and 18 577 women who were normotensive in both first and second pregnancy. Women with repeat preeclampsia had higher systolic and diastolic blood pressure, increased risk of hypertension and higher BMI, waist circumference, and serum glucose in midlife compared with women with only 1 occurrence of preeclampsia (Figures S7 through S10). Women with repeat preeclampsia also tended to have more adverse levels of all other cardiovascular risk factors except estimated glomerular filtration rate, but the low number of women with repeat preeclampsia precluded precise estimates. Life course trajectory of diabetes mellitus prevalence among women with repeat preeclampsia could not be estimated because of too few events.

## Discussion

In this longitudinal population‐based study, multiple cardiovascular risk factors were already elevated before first pregnancy in women who later experienced HDP compared with women with normotensive first pregnancies. Risk factor trajectories of women with HDP and normotensive first pregnancy displayed a roughly parallel pattern after pregnancy, but the increases in systolic blood pressure and measures of adiposity from 20 to 60 years of age were somewhat steeper among women with HDP. Although levels of blood pressure, adiposity, serum lipids, and glucose increased with age in both groups of women, there was a time lag of 10 years or more between mean levels observed among women with a history of HDP and women with normotensive first pregnancies. The time‐related cardiovascular risk profiles were similar in women with preeclampsia and gestational hypertension. Women with repeat preeclampsia in their first and second pregnancy had a more adverse cardiovascular risk factor profile than women with only 1 occurrence of preeclampsia in their first 2 pregnancies.

In our previous analysis on parity and life course blood pressure trajectories from this cohort, we observed that women with HDP as a group had higher blood pressure from before first pregnancy until beyond 60 years of age.^18^ In the present study, we examined a wide range of cardiovascular risk factors separately among women with preeclampsia and gestational hypertension. We are not aware of other studies that have constructed and contrasted life course trajectories of common cardiovascular risk factors in women with a history of HDP and women with normotensive pregnancies. Our work builds on previous studies by Magnussen et al, who examined the associations between pre‐ and postpregnancy cardiovascular risk factors and HDP; however, those studies were restricted to data from the HUNT1 and HUNTT2 surveys.^6^, ^9^, ^38^

Our results were generally consistent with previous studies in showing that women with HDP had adverse levels of cardiovascular risk factors at various time points from before first pregnancy and until menopause,^6^, ^7^, ^8^, ^9^, ^10^, ^11^, ^12^, ^13^, ^14^, ^15^, ^16^, ^17^ with correspondingly increased risks of hypertension, obesity, and diabetes mellitus.^13^, ^14^, ^15^, ^39^, ^40^, ^41^, ^42^ Our study adds to the limited evidence beyond age 50 years, confirming that except for lipids, for which trajectories converge by age 60 years, other differences in cardiovascular risk factors persist until age 60 years. Our findings also support the theoretical cardiovascular risk factor trajectories in women with HDP proposed by Sattar and Greer,^3^ giving credence to the concept of pregnancy as a stress test of cardiometabolic function. Additionally, the observation that most cardiovascular risk factors increase nearly monotonically with advancing age in women is also consistent with previous life course trajectory studies on selected cardiovascular risk factors.^43^

We were able to describe risk factor trajectories in normotensive and HDP women with high precision and with a longer follow‐up than previous studies, by applying mixed‐effects models.^32^ The use of repeated observations of cardiovascular risk factors pre‐ and post–first pregnancy was one of the major advantages of our study over previous ones, enabling the estimation of within‐woman trajectories and hence the ability to assess when higher levels of cardiovascular risk factors in HDP women were present. Our sensitivity analyses among women with 2 or more observations only confirmed that the trajectories including the full sample can be interpreted as within‐woman life course trajectories.

Our aim was to describe and contrast life course trajectories of cardiovascular risk factors in order to inform CVD screening and ultimately prevention in women with HDP. For that purpose, confounder adjustment was less relevant compared with studies aiming to examine the causal association of cardiovascular risk factors with HDP. Nevertheless, we adjusted for educational level and smoking, which are well established and easily identified prepregnancy factors potentially part of a common cause of HDP and cardiovascular risk factor elevation. Prepregnancy BMI may also be part of this common cause, but incomplete information prevented us from examining the impact of prepregnancy BMI on the life course trajectories. We adjusted for age and HUNT survey occasion, which should reduce the potential impact that secular trends in blood pressure,^44^ BMI,^45^ waist circumference,^46^ and cholesterol^47^ during our study period may have had on the observed difference between HDP and normotensive women. Antihypertensive treatment was used more frequently in women with a history of HDP, and although we attempted to remedy this by adding constants to the observed blood pressure measurements, as recommended by Cui et al^26^ and Tobin et al,^27^ antihypertensive use could have lowered blood pressure in HDP more than in normotensive women and attenuated the estimated difference between the groups. The use of statin treatment has increased substantially in Norway starting in the late 1990s^48^ and could have lowered non‐HDL cholesterol levels in women attending HUNT3 (2006–2008). In a similar way, the use of β‐blockers could have lowered the resting heart rate of women with HDP to a larger extent than for women without HDP. This may have contributed to the smaller differences in non‐HDL cholesterol levels and resting heart rate between HDP and normotensive women who we observed after 50 years of age, when statin and β‐blocker use is more frequent.

Participation declined in the more recent HUNT surveys and was lower among people with lower socioeconomic status and certain adverse health outcomes. However, the use of antihypertensive medication was similar in participants and nonparticipants,^49^ and nonparticipants had lower BMI than participants.^49^ It also seems unlikely that participation was related to HDP. For these reasons we do not expect nonparticipation to have violated the missing at random assumption implicit in mixed effects models nor caused substantial bias in the differences in cardiovascular risk factors between normotensive and HDP women. The MBRN provided accurate information on the reproductive histories, and the validity of the preeclampsia diagnosis within this population was generally good with a positive predictive value of 88%.^23^ For gestational hypertension, the positive predictive value was 68%, but most women with an MBRN diagnosis of gestational hypertension had evidence of either gestational hypertension or preeclampsia in medical records.^23^

The absence of noticeable differences between cardiovascular risk factor profiles in women with preeclampsia and gestational hypertension could in part be explained by most (84%) of the women diagnosed with preeclampsia having a mild form, as indicated by term delivery (gestational length ≥37 weeks). We did not have a sufficient number of women with preterm preeclampsia to examine whether this form of preeclampsia was associated with different cardiovascular risk trajectories. A validation study^23^ conducted within the same cohort also noted that some women diagnosed with gestational hypertension displayed signs of preeclampsia (ie, proteinuria), a finding that indicates overlap between the 2 groups of women.

Our and others’ observations that women with subsequent HDP have adverse cardiovascular risk factors in young adult life, before first pregnancy, support the hypothesis that adverse cardiovascular risk profiles observed in women with HDP originate early in life. These findings could be consistent with a genetic origin of HDP, but while the familial clustering of preeclampsia is well documented,^50^ there is limited knowledge about a possible genetic basis for the disorder.^51^ The higher risk of HDP in women who were born prematurely or with low birthweight^52^ supports that the elevated cardiovascular risk factor levels in women with HDP may be attributed to genes or to adverse in utero conditions.^53^ Alternatively, women who go on to develop HDP may have different dietary and lifestyle patterns in childhood and adolescence that set them on a divergent adult cardiovascular risk factor trajectory.

Although women with subsequent HDP have an adverse cardiovascular risk factor profile even before first pregnancy, this does not exclude an additional causal contribution by HDP.^3^ However, pre‐ to postpregnancy changes in most cardiovascular risk factors were similar between women with HDP and normotensive women, suggesting that HDP itself did not contribute to the adverse levels of these risk factors. The observation that BMI increased more in pregnancies with HDP is consistent with previous findings of increased risk of HDP with higher gestational weight gain,^54^ but it does not imply that HDP necessarily caused the higher pre‐ to postpregnancy increase in BMI.

As expected from the higher BMI, blood pressure, and glucose levels in women with HDP, the prevalence of obesity, hypertension, and diabetes mellitus remained elevated in women with HDP compared with women with normotensive pregnancies for the entire age range of 20 to 60 years. From a clinical perspective, it may be interesting to note that the probability of hypertension in preeclamptic women started increasing more rapidly at around age 30, approximately a decade earlier than in normotensive women, creating a time lag in the prevalence of hypertension of around 10 years. Obesity, hypertension, and diabetes mellitus are well known to increase the risk of CVD.^55^ Given the substantial body of evidence showing higher levels of cardiovascular risk factors in women with HDP, it is highly likely that a substantial proportion of the excess CVD risk in women with HDP^56^ is mediated through these traditional cardiovascular risk factors.

Research suggests that a reduction of 2 mm Hg in diastolic blood pressure could reduce the risk of coronary heart disease by 6% and the risk of stroke and transient ischemic attacks by 15%.^57^ Even such small reductions in blood pressure as that obtainable by lifestyle modification programs could be beneficial in women with a history of HDP. As the adverse cardiovascular risk profile in women with a history of HDP in most cases is already established in early adulthood, our findings suggest that HDP may be included in early CVD screening, and that women with HDP may particularly benefit from early lifestyle modification programs that target cardiometabolic risk factors following a pregnancy complicated by HDP.

## Conclusion

This longitudinal population‐based study shows that the adverse cardiovascular risk factor profiles in women with HDP are present before first pregnancy and remain higher compared with other women beyond 50 years of age. Progression of cardiovascular risk factors throughout the age interval 20 to 60 years occurs mostly in parallel for women with and without a history of HDP, with greater increases in systolic blood pressure and adiposity in women with a history of HDP. Women with a history of HDP may be expected to pass beyond treatment thresholds of blood pressure, adiposity, serum lipids, and glucose at least 10 years earlier than women with normotensive pregnancy. HDP signals long‐term increases in modifiable cardiovascular risk factors that may warrant early screening and preventive efforts.

## Sources of Funding

This work was supported by the Research Council of Norway (grant number 231149/F20) to Åsvold, Horn, and Haug. Åsvold was also supported by the Liaison Committee for Education, Research and Innovation in Central Norway, and by the Fulbright Program. Fraser is supported by a personal fellowship from the UK Medical Research Council (MRC) (grant number MR/M009351/1). Fraser and Tilling work in a Unit that receives core funding from Medical Research Council in the UK (grant number MC_UU_12013/5). This work was also supported by the American Heart Association (grant number 16PRE29690006) to Markovitz.

## Disclosures

Disclosures are correct.

## Supporting information


**Table S1.** Number of Women and Measurements Included in Analysis by CVD Risk Factor
**Table S2.** Predicted Mean Levels of CVD Risk Factors by Age at Follow‐Up in Women With Normotensive and Preeclamptic First Pregnancies
**Table S3.** Predicted Change in CVD Risk Factor Level From Pre‐ to Post–First Pregnancy in Women With Normotensive or Preeclamptic First Pregnancy
**Table S4.** Predicted Change Per Year in CVD Risk Factors by Age Interval in Women With Normotensive and Preeclamptic First Pregnancies
**Table S5.** Population Average Predicted Probabilities* of Hypertension, Obesity, and Diabetes Mellitus by Age at Follow‐Up in Women With Normotension, Preeclampsia, and Gestational Hypertension in First Pregnancy
**Figure S1.** Number (A) and proportion (B) of HUNT participants according to age at participation and HUNT survey.
**Figure S2.** Life course trajectories of mean systolic blood pressure (A), diastolic blood pressure (B), BMI (C), waist circumference (D), hip circumference (E), and waist‐to‐hip ratio (F) for women with normotension and gestational hypertension in their first pregnancies.
**Figure S3.** Life course trajectories of mean nonfasting non‐HDL (A) and HDL (B) cholesterol, triglycerides (C), and glucose (D), resting heart rate (E), and serum CRP (F) for women with normotension and gestational hypertension in their first pregnancies.
**Figure S4.** Life course trajectories of mean estimated glomerular filtration rate (eGFR) for women with normotension, preeclampsia (A), or gestational hypertension (B) in their first pregnancies.
**Figure S5.** Life course trajectories of mean systolic blood pressure (A), diastolic blood pressure (B), BMI (C), waist circumference (D), hip circumference (E), and waist‐to‐hip ratio (F) for women with normotensive and preeclamptic first pregnancies who had 2 or more observations.
**Figure S6.** Life course trajectories of mean nonfasting serum non‐HDL (A) and HDL (B) cholesterol, triglycerides (C) and glucose (D), resting heart rate (E), and estimated glomerular filtration rate (F) for women with normotensive and preeclamptic first pregnancies who had 2 or more observations.
**Figure S7.** Life course trajectories of mean systolic blood pressure (A), diastolic blood pressure (B), BMI (C), waist circumference (D), hip circumference (E), and waist‐to‐hip ratio (F) for women with normotensive first and second pregnancy, preeclampsia in 1 of their first 2 pregnancies, and preeclampsia in both the first and second pregnancy.
**Figure S8.** Life course trajectories of mean nonfasting serum non‐HDL (A) and HDL (B) cholesterol, triglycerides (C) and glucose (D), resting heart rate (E), and serum CRP (F) for women with normotensive first and second pregnancy, preeclampsia in 1 of their first 2 pregnancies, and preeclampsia in both the first and second pregnancy.
**Figure S9.** Life course trajectories of mean estimated glomerular filtration rate (eGFR) for women with normotensive first and second pregnancy, preeclampsia in 1 of their first 2 pregnancies, and preeclampsia in both the first and second pregnancy.
**Figure S10.** Population average predicted probabilities of hypertension (defined as current antihypertensive medication and/or blood pressure ≥140 mm Hg systolic or ≥90 mm Hg diastolic) (A) and obesity (defined as a BMI ≥30 kg/m^2^) (B) by age in women with normotensive first and second pregnancy, preeclampsia in 1 of their first 2 pregnancies, and preeclampsia in both the first and second pregnancy.
