# Supplementary material for: Life Course Trajectories of Cardiovascular Risk Factors in Women With and Without Hypertensive Disorders in First Pregnancy: The HUNT Study in Norway
Source: J Am Heart Assoc. 2018 Jul 27;7(15):e009250. doi: 10.1161/JAHA.118.009250 (PMC6201453; doi:10.1161/JAHA.118.009250)
Supplement: Supplementary file 1 — Table S1. Number of Women and Measurements Included in Analysis by CVD Risk Factor Table S2. Predicted Mean Levels of CVD Risk Factors by Age at Follow‐Up in Women With Normotensive and Preeclamptic First Pregnancies Table S3. Predicted Change in CVD Risk Factor Level From Pre‐ to Post–First Pregnancy in Women With Normotensive or Preeclamptic First Pregnancy Table S4. Predicted Change Per Year in CVD Risk Factors by Age Interval in Women With Normotensive and Preeclamptic First Pregnancies Table S5. Population Average Predicted Probabilities* of Hypertension, Obesity, and Diabetes Mellitus by Age at Follow‐Up in Women With Normotension, Preeclampsia, and Gestational Hypertension in First Pregnancy Figure S1. Number (A) and proportion (B) of HUNT participants according to age at participation and HUNT survey. Figure S2. Life course trajectories of mean systolic blood pressure (A), diastolic blood pressure (B), BMI (C), waist circumference (D), hip circumference (E), and waist‐to‐hip ratio (F) for women with normotension and gestational hypertension in their first pregnancies. Figure S3. Life course trajectories of mean nonfasting non‐HDL (A) and HDL (B) cholesterol, triglycerides (C), and glucose (D), resting heart rate (E), and serum CRP (F) for women with normotension and gestational hypertension in their first pregnancies. Figure S4. Life course trajectories of mean estimated glomerular filtration rate (eGFR) for women with normotension, preeclampsia (A), or gestational hypertension (B) in their first pregnancies. Figure S5. Life course trajectories of mean systolic blood pressure (A), diastolic blood pressure (B), BMI (C), waist circumference (D), hip circumference (E), and waist‐to‐hip ratio (F) for women with normotensive and preeclamptic first pregnancies who had 2 or more observations. Figure S6. Life course trajectories of mean nonfasting serum non‐HDL (A) and HDL (B) cholesterol, triglycerides (C) and glucose (D), resting heart rate (E), and estimated glom [file JAH3-7-e009250-s001.pdf]

SUPPLEMENTAL MATERIAL

Table S1. Number of women and measurements included in analysis by CVD risk factor.

CVD risk factor	Number of women			Number of measurements		
	Normotension	Preeclampsia	Gestational hypertension	Normotension	Preeclampsia	Gestational hypertension
Systolic blood pressure	22 061	1077	474	42 357	1976	947
Diastolic blood pressure	22 061	1077	474	42 356	1976	947
BMI	22 298	1091	478	54 422	2519	1177
Waist circumference	20 409	1009	433	31 361	1510	678
Hip circumference	20 410	1009	433	31 362	1510	678
Waist to hip ratio	20 409	1009	433	31 360	1510	678
Non-HDL cholesterol	20 283	1007	432	30 977	1493	668
HDL cholesterol	20 283	1007	432	30 977	1493	668
Triglycerides	19 858	977	427	30 715	1470	666
Glucose	19 836	983	429	31 156	1510	683
Resting heart rate	21 530	1049	467	40 406	1875	895
CRP	16 335	791	330	17 983	885	370

Table S2. Predicted mean levels of cardiovascular disease risk factors by age at follow-up in women with normotensive and preeclamptic first pregnancies.

	Normotensive		Preeclampsia		Difference		
**Linear prediction***	estimate	95% CI	estimate	95% CI	estimate	95% CI	p-value
**Systolic blood pressure (mmHg)**							
20 years	119.03	[118.33 – 119.73]	124.23	[122.21 – 126.24]	5.20	[3.20 – 7.20]	<0.001
1st birth occurs at age 23							
30 years	116.95	[116.62 – 117.29]	123.10	[121.78 – 124.42]	6.15	[4.81 – 7.48]	<0.001
40 years	121.08	[120.79 – 121.38]	127.20	[125.85 – 128.56]	6.12	[4.74 – 7.50]	<0.001
50 years	129.32	[128.91 – 129.72]	138.69	[136.90 – 140.49]	9.38	[7.55 – 11.20]	<0.001
60 years	137.72	[137.03 – 138.41]	146.72	[143.94 – 149.49]	8.99	[6.20 – 11.79]	<0.001
**Diastolic blood pressure (mmHg)**							
20 years	68.03	[67.50 – 68.57]	71.49	[69.97 – 73.02]	3.46	[1.95 – 4.97]	<0.001
30 years	69.99	[69.75 – 70.23]	75.15	[74.20 – 76.10]	5.16	[4.20 – 6.12]	<0.001
40 years	74.29	[74.09 – 74.49]	78.63	[77.71 – 79.54]	4.33	[3.40 – 5.27]	<0.001
50 years	78.61	[78.35 – 78.87]	83.82	[82.66 – 84.98]	5.21	[4.03 – 6.38]	<0.001
60 years	79.86	[79.43 – 80.30]	82.68	[80.91 – 84.45]	2.82	[1.03 – 4.60]	0.002
**BMI (kg/m^2)**							
20 years	22.78	[22.69 – 22.86]	23.82	[23.61 – 24.04]	1.05	[0.84 – 1.26]	<0.001
30 years	23.92	[23.83 – 24.00]	26.25	[25.95 – 26.56]	2.34	[2.03 – 2.65]	<0.001
40 years	24.89	[24.81 – 24.97]	26.97	[26.64 – 27.30]	2.08	[1.75 – 2.41]	<0.001
50 years	25.92	[25.81 – 26.03]	28.36	[27.95 – 28.77]	2.44	[2.03 – 2.85]	<0.001
60 years	26.62	[26.45 – 26.79]	29.01	[28.44 – 29.58]	2.39	[1.82 – 2.96]	<0.001
**Waist circumference (cm)**							
20 years	77.08	[76.39 – 77.78]	79.82	[77.93 – 81.72]	2.74	[0.86 – 4.62]	0.004
30 years	81.79	[81.44 – 82.13]	86.88	[85.67 – 88.10]	5.09	[3.86 – 6.33]	<0.001
40 years	83.35	[83.11 – 83.60]	87.99	[86.92 – 89.06]	4.64	[3.55 – 5.73]	<0.001
50 years	85.37	[85.10 – 85.63]	90.49	[89.28 – 91.69]	5.12	[3.89 – 6.35]	<0.001
60 years	86.69	[86.26 – 87.11]	90.43	[88.72 – 92.13]	3.74	[2.02 – 5.46]	<0.001
**Hip circumference (cm)**							
20 years	97.98	[97.35 – 98.60]	98.91	[97.19 – 100.63]	0.93	[-0.77 – 2.63]	0.281
30 years	100.80	[100.50 – 101.09]	105.53	[104.49 – 106.57]	4.73	[3.68 – 5.79]	<0.001
40 years	101.88	[101.68 – 102.07]	105.15	[104.29 – 106.02]	3.28	[2.40 – 4.16]	<0.001
50 years	102.83	[102.63 – 103.04]	106.03	[105.10 – 106.97]	3.20	[2.25 – 4.15]	<0.001
60 years	102.72	[102.40 – 103.04]	105.09	[103.79 – 106.38]	2.37	[1.06 – 3.67]	<0.001
**Waist to hip ratio**							
20 years	0.78	[0.78 – 0.79]	0.80	[0.79 – 0.81]	0.02	[0.01 – 0.03]	0.001
30 years	0.81	[0.81 – 0.81]	0.82	[0.82 – 0.83]	0.01	[0.01 – 0.02]	0.001
40 years	0.82	[0.81 – 0.82]	0.83	[0.83 – 0.84]	0.02	[0.01 – 0.02]	<0.001
50 years	0.83	[0.83 – 0.83]	0.85	[0.84 – 0.86]	0.02	[0.01 – 0.03]	<0.001
60 years	0.84	[0.84 – 0.84]	0.85	[0.84 – 0.87]	0.01	[0.00 – 0.02]	0.045

*Linear predictions are estimated with all covariates set at their means and as if the woman has her first birth at age 23.

Table S2 continued. Predicted mean levels of cardiovascular disease risk factors by age at follow-up in women with normotensive and preeclamptic first pregnancies.

	**Normotensive**		**Preeclampsia**		**Difference**		
**Linear prediction***	estimate	95% CI	estimate	95% CI	estimate	95% CI	p-value
**Non-HDL cholesterol (mmol/L)**							
20 years	3.18	[3.11 – 3.24]	3.42	[3.22 – 3.61]	0.24	[0.05 – 0.43]	0.013
		1st birth occurs at age 23					
30 years	3.40	[3.37 – 3.44]	3.64	[3.52 – 3.76]	0.24	[0.11 – 0.36]	<0.001
40 years	3.72	[3.70 – 3.74]	3.88	[3.78 – 3.99]	0.16	[0.05 – 0.27]	0.003
50 years	4.31	[4.29 – 4.34]	4.42	[4.30 – 4.54]	0.11	[-0.01 – 0.23]	0.073
60 years	4.77	[4.73 – 4.81]	4.73	[4.56 – 4.90]	-0.04	[-0.21 – 0.13]	0.663
**HDL cholesterol (mmol/L)**							
20 years	1.43	[1.41 – 1.46]	1.44	[1.38 – 1.51]	0.01	[-0.05 – 0.08]	0.724
30 years	1.39	[1.38 – 1.40]	1.32	[1.28 – 1.36]	-0.07	[-0.11 – -0.03]	0.001
40 years	1.43	[1.42 – 1.44]	1.37	[1.34 – 1.41]	-0.06	[-0.10 – -0.03]	0.001
50 years	1.53	[1.52 – 1.54]	1.50	[1.46 – 1.54]	-0.03	[-0.07 – 0.01]	0.175
60 years	1.55	[1.54 – 1.56]	1.55	[1.49 – 1.60]	0.00	[-0.06 – 0.05]	0.923
**Triglycerides (mmol/L)**							
20 years	1.22	[1.170 – 1.269]	1.40	[1.266 – 1.540]	0.18	[0.05 – 0.32]	0.008
30 years	1.16	[1.13 – 1.18]	1.29	[1.188 – 1.384]	0.13	[0.03 – 0.23]	0.011
40 years	1.23	[1.21 – 1.25]	1.39	[1.30 – 1.48]	0.16	[0.06 – 0.25]	0.001
50 years	1.45	[1.42 – 1.47]	1.58	[1.48 – 1.69]	0.14	[0.03 – 0.24]	0.009
60 years	1.67	[1.63 - 1.70]	1.70	[1.55 - 1.86]	0.04	[-0.12 – 0.19]	0.649
**Glucose (mmol/L)**							
20 years	4.87	[4.79 – 4.95]	5.07	[4.85 – 5.28]	0.20	[-0.01 – 0.41]	0.068
30 years	4.89	[4.85 – 4.93]	5.07	[4.94 – 5.20]	0.18	[0.04 – 0.32]	0.010
40 years	5.12	[5.09 – 5.15]	5.19	[5.07 – 5.31]	0.07	[-0.05 – 0.19]	0.268
50 years	5.32	[5.29 – 5.35]	5.47	[5.32 – 5.61]	0.14	[-0.00 – 0.29]	0.052
60 years	5.58	[5.53 – 5.63]	5.80	[5.56 – 6.04]	0.22	[-0.02 – 0.46]	0.076
**Resting heart rate (beats/min)**							
20 years	75.07	[74.38 – 75.76]	77.45	[75.48 – 79.42]	2.38	[0.42 – 4.33]	0.017
30 years	73.90	[73.60 – 74.20]	74.94	[73.74 – 76.13]	1.03	[-0.18 – 2.25]	0.095
40 years	73.63	[73.38 – 73.87]	74.56	[73.45 – 75.67]	0.93	[-0.20 – 2.06]	0.105
50 years	72.96	[72.65 – 73.27]	74.26	[72.88 – 75.64]	1.30	[-0.10 – 2.70]	0.068
60 years	73.12	[72.61 – 73.63]	71.50	[69.39 – 73.62]	-1.61	[-3.75 – 0.53]	0.139
**CRP† (mg/L)**							
20 years	1.69	[1.45 – 1.97]	1.69	[1.08 – 2.63]	1.00	[0.64 – 1.56]	0.997
30 years	1.00	[0.93 – 1.07]	1.15	[0.89 – 1.49]	1.15	[0.88 – 1.50]	0.297
40 years	0.81	[0.78 – 0.85]	1.05	[0.89 – 1.23]	1.29	[1.09 – 1.53]	0.003
50 years	0.96	[0.92 – 1.00]	1.25	[1.04 – 1.49]	1.30	[1.08 – 1.56]	0.005
60 years	1.30	[1.24 – 1.36]	1.40	[1.14 – 1.72]	1.08	[0.87 – 1.33]	0.487

*Linear predictions are estimated with all covariates set at their means and as if the woman has her first birth at age 23.
†CRP is given as geometric mean values where the difference equates to the ratio of geometric mean CRP between women with preeclamptic and normotensive first pregnancy.

Table S3. Predicted change in cardiovascular disease risk factor level from pre- to post-first pregnancy in women with normotensive or preeclamptic first pregnancy.

	Normotension		Preeclampsia		Difference		
	Change	95 % CI	Change	95 % CI	Change	95 % CI	p-value
**Systolic blood pressure (mmHg)**	-3.81	[-4.65 – -2.97]	-2.99	[-5.32 – -0.67]	0.82	[-1.47 – 3.11]	0.485
**Diastolic blood pressure (mmHg)**	-2.17	[-2.81 – -1.53]	-0.44	[-2.17 – 1.29]	1.72	[0.03 – 3.42]	0.046
**BMI (kg/m^2)**	0.03	[-0.08 – 0.15]	0.95	[0.51 – 1.40]	0.92	[0.46 – 1.37]	<0.001
**Waist circumference (cm)**	3.06	[2.19 – 3.93]	5.21	[2.86 – 7.57]	2.15	[-0.17 – 4.47]	0.070
**Hip circumference (cm)**	1.03	[0.24 – 1.81]	2.29	[0.21 – 4.38]	1.27	[-0.78 – 3.31]	0.226
**Waist to hip ratio**	0.02	[0.02 – 0.03]	0.03	[0.01 – 0.04]	0.00	[-0.01 – 0.02]	0.549
**Non-HDL cholesterol (mmol/L)**	-0.02	[-0.10 – 0.07]	0.09	[-0.14 – 0.33]	0.11	[-0.12 – 0.34]	0.363
**HDL cholesterol (mmol/L)**	-0.14	[-0.17 – -0.11]	-0.11	[-0.19 – -0.03]	0.02	[-0.06 – 0.10]	0.567
**Triglycerides (mmol/L)**	0.01	[-0.06 – 0.07]	0.05	[-0.12 – 0.23]	0.04	[-0.13 – 0.22]	0.614
**Glucose (mmol/L)**	-0.10	[-0.20 – -0.01]	-0.32	[-0.58 – -0.07]	-0.22	[-0.47 – 0.03]	0.090
**Resting heart rate (beats/min)**	0.94	[0.11 – 1.77]	0.44	[-1.77 – 2.65]	-0.50	[-2.66 – 1.66]	0.649
**CRP* (mg/L)**	0.85	[0.69 – 1.05]	0.79	[0.45 – 1.39]	0.93	[0.53 – 1.63]	0.797

*CRP is given as geometric mean values where the difference equates to the ratio of geometric mean CRP between women with preeclampsia and normotensive women.

Table S4. Predicted change per year in cardiovascular disease risk factors by age interval in women with normotensive and preeclamptic first pregnancies.

	Normotension		Preeclampsia		Difference		
**Change per year**	estimate	95% CI	estimate	95% CI	estimate	95% CI	p-value
**Systolic blood pressure (mmHg/year)**							
20-23 years	0.09	[-0.04 – 0.22]	0.38	[0.03 – 0.73]	0.29	[-0.05 – 0.63]	0.097
23-30 years	0.21	[0.12 – 0.30]	0.09	[-0.25 – 0.43]	-0.12	[-0.46 – 0.23]	0.500
30-40 years	0.43	[0.37 – 0.50]	0.41	[0.21 – 0.61]	0.00	[-0.21 –0.20]	0.981
40-50 years	0.84	[0.79 – 0.89]	1.15	[0.93 – 1.36]	0.33	[0.11 – 0.54]	0.004
50-60 years	0.84	[0.77 – 0.91]	0.80	[0.50 – 1.11]	-0.04	[-0.35 – 0.27]	0.811
**Diastolic blood pressure (mmHg/year)**							
20-23 years	0.44	[0.34 – 0.54]	0.62	[0.36 – 0.87]	0.17	[-0.07 – 0.42]	0.170
23-30 years	0.40	[0.33 – 0.47]	0.31	[0.06 – 0.56]	-0.09	[-0.34 – 0.16]	0.489
30-40 years	0.44	[0.39 – 0.48]	0.35	[0.21 – 0.49]	-0.08	[-0.22 – 0.06]	0.249
40-50 years	0.44	[0.41 – 0.47]	0.52	[0.38 – 0.66]	0.09	[-0.06 – 0.23]	0.239
50-60 years	0.13	[0.08 – 0.17]	-0.11	[-0.32 – 0.09]	-0.24	[-0.45 – -0.03]	0.023
**BMI (kg/m^2/year)**							
18-23 years	0.08	[0.06 – 0.10]	0.16	[0.11 – 0.20]	0.08	[0.03 – 0.12]	0.001
23-30 years	0.13	[0.11 – 0.14]	0.14	[0.09 – 0.20]	0.02	[-0.03 – 0.07]	0.502
30-40 years	0.10	[0.09 – 0.12]	0.07	[0.04 – 0.11]	-0.03	[-0.06 – 0.01]	0.151
40-50 years	0.10	[0.09 – 0.11]	0.14	[0.10 – 0.17]	0.04	[-0.00 – 0.07]	0.052
50-60 years	0.07	[0.06 – 0.08]	0.07	[0.02 – 0.12]	0.00	[-0.06 – 0.05]	0.850
**Waist circumference (cm/year)**							
20-23 years	0.17	[0.05 – 0.29]	0.30	[-0.02 – 0.62]	0.13	[-0.18 – 0.45]	0.401
23-30 years	0.16	[0.06 – 0.26]	0.13	[-0.19 – 0.44]	-0.03	[-0.35 – 0.28]	0.835
30-40 years	0.18	[0.12 – 0.23]	0.11	[-0.05 – 0.27]	-0.05	[-0.21 – 0.11]	0.576
40-50 years	0.18	[0.15 – 0.21]	0.25	[0.10 – 0.39]	0.05	[-0.10 – 0.20]	0.522
50-60 years	0.13	[0.09 – 0.17]	-0.01	[-0.18 – 0.17]	-0.14	[-0.32 – 0.04]	0.135
**Hip circumference (cm/year)**							
20-23 years	0.18	[0.07 – 0.28]	0.48	[0.21 – 0.76]	0.31	[0.04 – 0.58]	0.027
23-30 years	0.18	[0.10 – 0.26]	0.41	[0.14 – 0.68]	0.23	[-0.05 – 0.50]	0.105
30-40 years	0.13	[0.09 – 0.18]	-0.04	[-0.17 – 0.09]	-0.15	[-0.28 – -0.01]	0.032
40-50 years	0.09	[0.06 – 0.12]	0.09	[-0.03 – 0.21]	-0.01	[-0.13 – 0.11]	0.899
50-60 years	-0.01	[-0.04 - 0.02]	-0.09	[-0.24 – 0.05]	-0.08	[-0.23 – 0.06]	0.258

Table S4 continued. Predicted change per year in cardiovascular disease risk factors by age interval in women with normotensive and preeclamptic first pregnancies.

	Normotension		Preeclampsia		Difference		
**Change per year**	estimate	95% CI	estimate	95% CI	estimate	95% CI	p-value
**Non-HDL cholesterol (mmol/L/year)**							
20-23 years	0.03	[0.02 – 0.04]	0.02	[-0.01 – 0.05]	-0.01	[-0.04 – 0.02]	0.647
23-30 years	0.02	[0.01 – 0.03]	0.01	[-0.02 – 0.04]	-0.01	[-0.05 – 0.02]	0.442
30-40 years	0.03	[0.02 – 0.04]	0.02	[0.01 – 0.04]	-0.01	[-0.02 – 0.01]	0.410
40-50 years	0.06	[0.06 – 0.06]	0.05	[0.04 – 0.07]	-0.01	[-0.02 – 0.01]	0.514
50-60 years	0.05	[0.04 – 0.05]	0.03	[0.01 – 0.05]	-0.01	[-0.03 – 0.01]	0.150
**HDL cholesterol (mmol/L/year)**							
20-23 years	0.01	[0.01 – 0.02]	0.00	[-0.01 – 0.01]	-0.01	[-0.02 – -0.00]	0.024
23-30 years	0.01	[0.01 – 0.01]	0.00	[-0.01 – 0.01]	-0.01	[-0.02 – 0.00]	0.085
30-40 years	0.00	[0.00 – 0.01]	0.00	[-0.00 – 0.01]	0.00	[-0.00 – 0.01]	0.780
40-50 years	0.01	[0.01 – 0.01]	0.01	[0.01 – 0.02]	0.00	[-0.00 – 0.01]	0.176
50-60 years	0.00	[0.00 – 0.00]	0.00	[-0.00 – 0.01]	0.00	[-0.00 – 0.01]	0.428
**Triglycerides (mmol/L/year)**							
20-23 years	-0.01	[-0.02 – -0.00]	-0.01	[-0.04 – 0.01]	0.00	[-0.03 – 0.02]	0.884
23-30 years	-0.01	[-0.01 – 0.00]	-0.02	[-0.04 – 0.01]	-0.01	[-0.04 – 0.01]	0.283
30-40 years	0.01	[0.00 – 0.01]	0.01	[-0.00 – 0.02]	0.00	[-0.01 – 0.02]	0.708
40-50 years	0.02	[0.02 – 0.02]	0.02	[0.01 – 0.03]	0.00	[-0.02 – 0.01]	0.804
50-60 years	0.02	[0.02 – 0.03]	0.01	[-0.01 – 0.03]	-0.01	[-0.03 – 0.01]	0.274
**Glucose (mmol/L/year)**							
20-23 years	0.01	[-0.00 – 0.03]	0.03	[-0.00 – 0.07]	0.02	[-0.01 – 0.06]	0.238
23-30 years	0.01	[0.00 – 0.02]	0.03	[-0.01 – 0.07]	0.02	[-0.02 – 0.06]	0.319
30-40 years	0.02	[0.02 – 0.03]	0.01	[-0.01 – 0.03]	-0.01	[-0.03 – 0.01]	0.286
40-50 years	0.02	[0.02 – 0.02]	0.03	[0.01 – 0.05]	0.01	[-0.01 – 0.03]	0.469
50-60 years	0.03	[0.02 – 0.03]	0.03	[0.01 – 0.06]	0.01	[-0.02 – 0.04]	0.609
**Resting heart rate (beats/min/year)**							
20-23 years	-0.13	[-0.26 – -0.01]	-0.12	[-0.44 – 0.21]	0.02	[-0.30 – 0.33]	0.920
23-30 years	-0.25	[-0.33 – -0.16]	-0.38	[-0.69 – -0.07]	-0.13	[-0.45 – 0.18]	0.409
30-40 years	-0.01	[-0.07 – 0.04]	-0.04	[-0.21 – 0.13]	-0.01	[-0.18 – 0.16]	0.909
40-50 years	-0.07	[-0.11 – -0.03]	-0.03	[-0.21 – 0.15]	0.04	[-0.14 – 0.22]	0.687
50-60 years	0.02	[-0.04 – 0.07]	-0.28	[-0.53 – -0.02]	-0.29	[-0.55 – -0.04]	0.026
**CRP* (mg/L/year)**							
20-23 years							
23-30 years	0.97	[0.94 – 1.00]	1.01	[0.93 – 1.09]	1.04	[0.97 – 1.12]	0.296
30-40 years	0.96	[0.94 – 0.99]	0.98	[0.91 – 1.05]	1.01	[0.94 – 1.09]	0.725
40-50 years	0.98	[0.97 – 0.99]	0.99	[0.96 – 1.02]	1.01	[0.98 – 1.05]	0.503
50-60 years	1.01	[1.01 – 1.02]	1.02	[0.99 – 1.04]	1.00	[0.97 – 1.03]	0.974

*CRP is given as geometric mean values where the difference equates to the ratio of geometric mean CRP between women with preeclampsia and normotensive women.

Table S5. Population average predicted probabilities* of hypertension, obesity and diabetes by age at follow-up in women with normotension, preeclampsia and gestational hypertension in first pregnancy.

	**First pregnancy**					
	**Normotension**		**Preeclampsia**		**Gestational hypertension**	
**Age**	probability	95% CI	probability	95% CI	probability	95% CI
**Hypertension**						
20 years	0.05	[0.04 – 0.06]	0.15	[0.10 – 0.23]	0.15	[0.07 – 0.29]
30 years	0.05	[0.04 – 0.06]	0.13	[0.10 – 0.17]	0.25	[0.18 – 0.32]
40 years	0.13	[0.12 – 0.13]	0.31	[0.26 – 0.36]	0.34	[0.28 – 0.41]
50 years	0.34	[0.32 – 0.35]	0.55	[0.49 – 0.62]	0.52	[0.43 – 0.60]
60 years	0.58	[0.55 – 0.60]	0.78	[0.70 – 0.84]	0.79	[0.69 – 0.87]
**Obesity**						
20 years	0.03	[0.02 , 0.03]	0.07	[0.04 , 0.10]	0.06	[0.03 , 0.11]
30 years	0.07	[0.06 , 0.08]	0.17	[0.13 , 0.21]	0.18	[0.13 , 0.25]
40 years	0.08	[0.08 , 0.09]	0.16	[0.13 , 0.20]	0.20	[0.16 , 0.26]
50 years	0.09	[0.08 , 0.10]	0.17	[0.13 , 0.21]	0.21	[0.15 , 0.28]
60 years	0.11	[0.10 , 0.13]	0.18	[0.12 , 0.24]	0.21	[0.13 , 0.31]
**Diabetes**						
20 years	0.003	[0.001 – 0.009]	0.02	[0.00 – 0.06]	0.000	[0.00 – 0.000]
30 years	0.004	[0.002 – 0.006]	0.01	[0.00 – 0.02]	0.015	[0.002 – 0.10]
40 years	0.01	[0.01 – 0.01]	0.02	[0.01 – 0.04]	0.002	[0.000 – 0.02]
50 years	0.01	[0.01 – 0.02]	0.04	[0.02 – 0.06]	0.02	[0.01 – 0.05]
60 years	0.03	[0.02 – 0.04]	0.06	[0.03 – 0.12]	0.10	[0.05 – 0.21]

*Population average proportions are estimated with all covariates set at their means and as if the woman has her first birth at age 23.

Figure S1. Number (a) and proportion (b) of HUNT participants according to age at participation and HUNT survey.

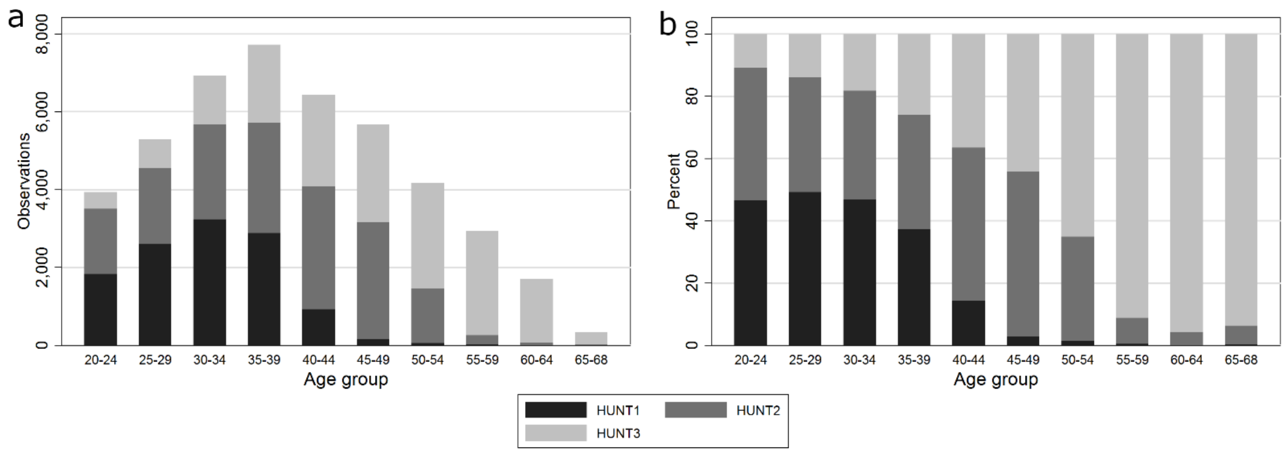

Figure S2. Life course trajectories of mean systolic blood pressure (a), diastolic blood pressure (b), BMI (c), waist circumference (d), hip circumference (e) and waist to hip ratio (f) for women with normotension and gestational hypertension in their first pregnancies.

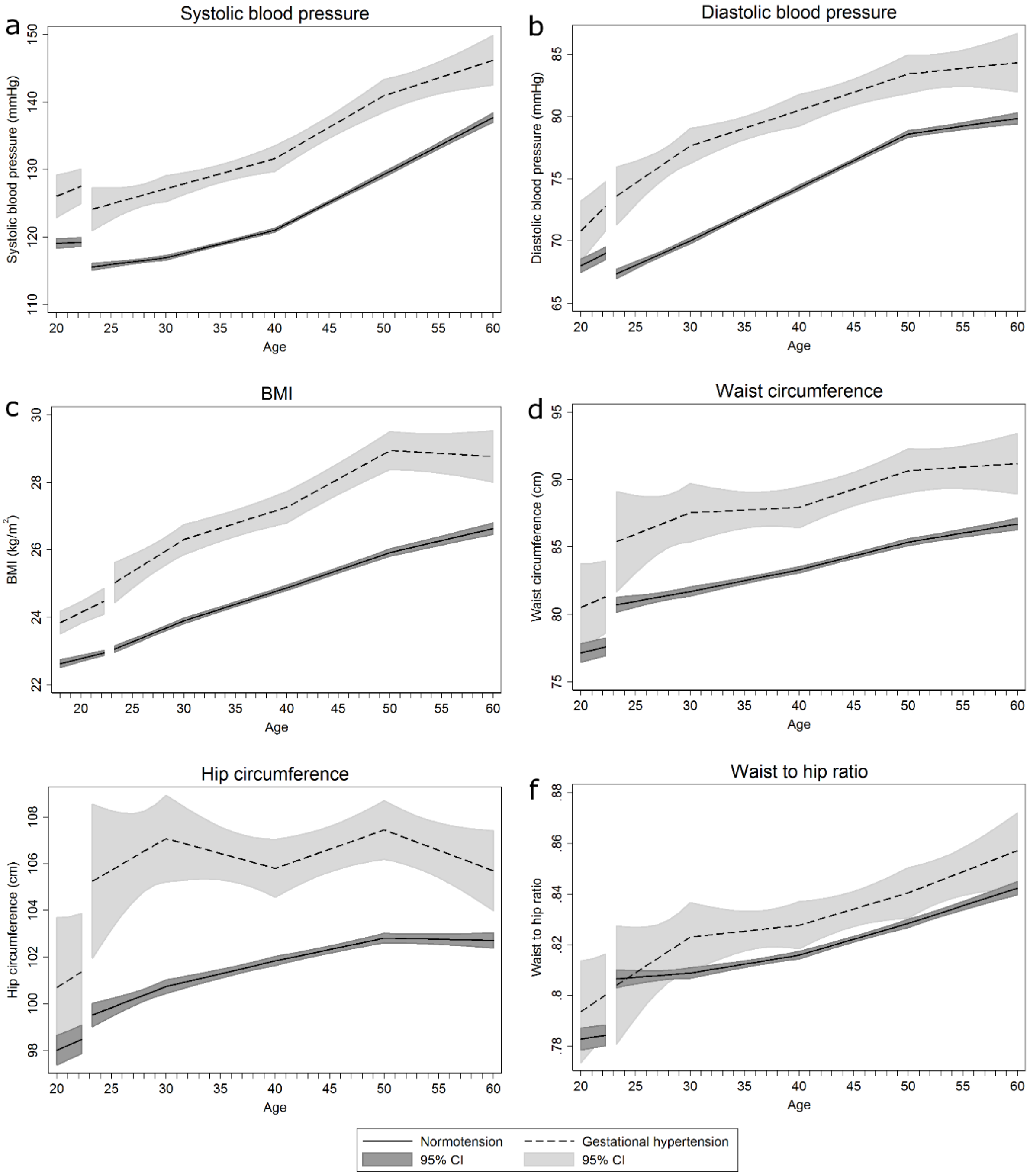

Estimates are adjusted for age at measurement, HUNT survey, highest obtained education level, age at first birth and ever daily smoking. Covariates are fixed at their means with gaps in the graphs corresponding to the woman's first pregnancy, birth at age 23 and a three-month postpartum period.

Figure S3. Life course trajectories of mean non-fasting non-HDL (a) and HDL (b) cholesterol, triglycerides (c), and glucose (d), resting heart rate (e), and serum CRP (f) for women with normotension and gestational hypertension in their first pregnancies.

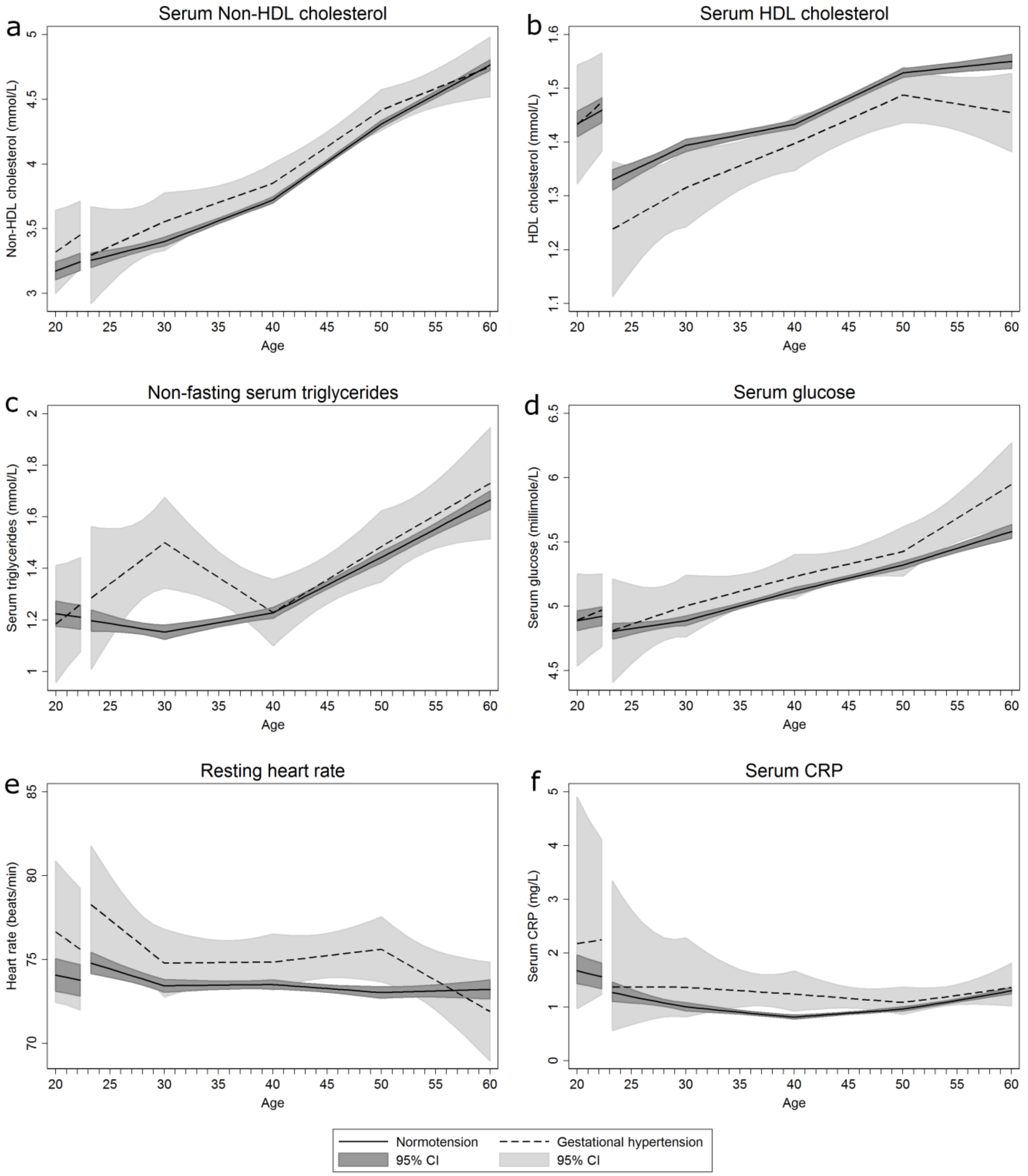

Estimates are adjusted for age at measurement, HUNT survey, highest obtained education level, age at first pregnancy and ever daily smoking. Analyses of glucose and triglycerides were additionally adjusted for time since last meal. Covariates are fixed at their means with gaps in the graphs corresponding to the woman's first pregnancy, birth at age 23 and a three-month postpartum period. CRP is given as geometric mean.

Figure S4. Life course trajectories of mean estimated glomerular filtration rate (eGFR) for women with normotension, preeclampsia (a) or gestational hypertension (b) in their first pregnancies.

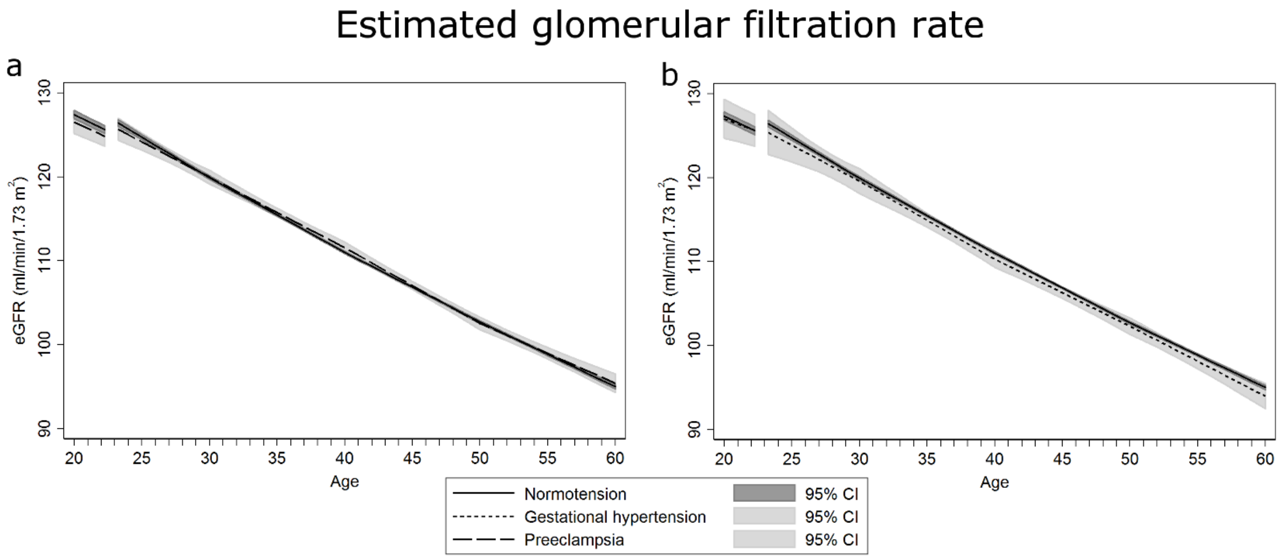

Estimates are adjusted for age at measurement, HUNT survey, highest obtained education level, age at first pregnancy and ever daily smoking. Covariates are fixed at their means with gaps in the graphs corresponding to the woman's first pregnancy, birth at age 23 and a three-month postpartum period.

Figure S5. Life course trajectories of mean systolic blood pressure (a), diastolic blood pressure (b), BMI (c), waist circumference (d), hip circumference (e) and waist to hip ratio (f) for women with normotensive and preeclamptic first pregnancies who had two or more observations.

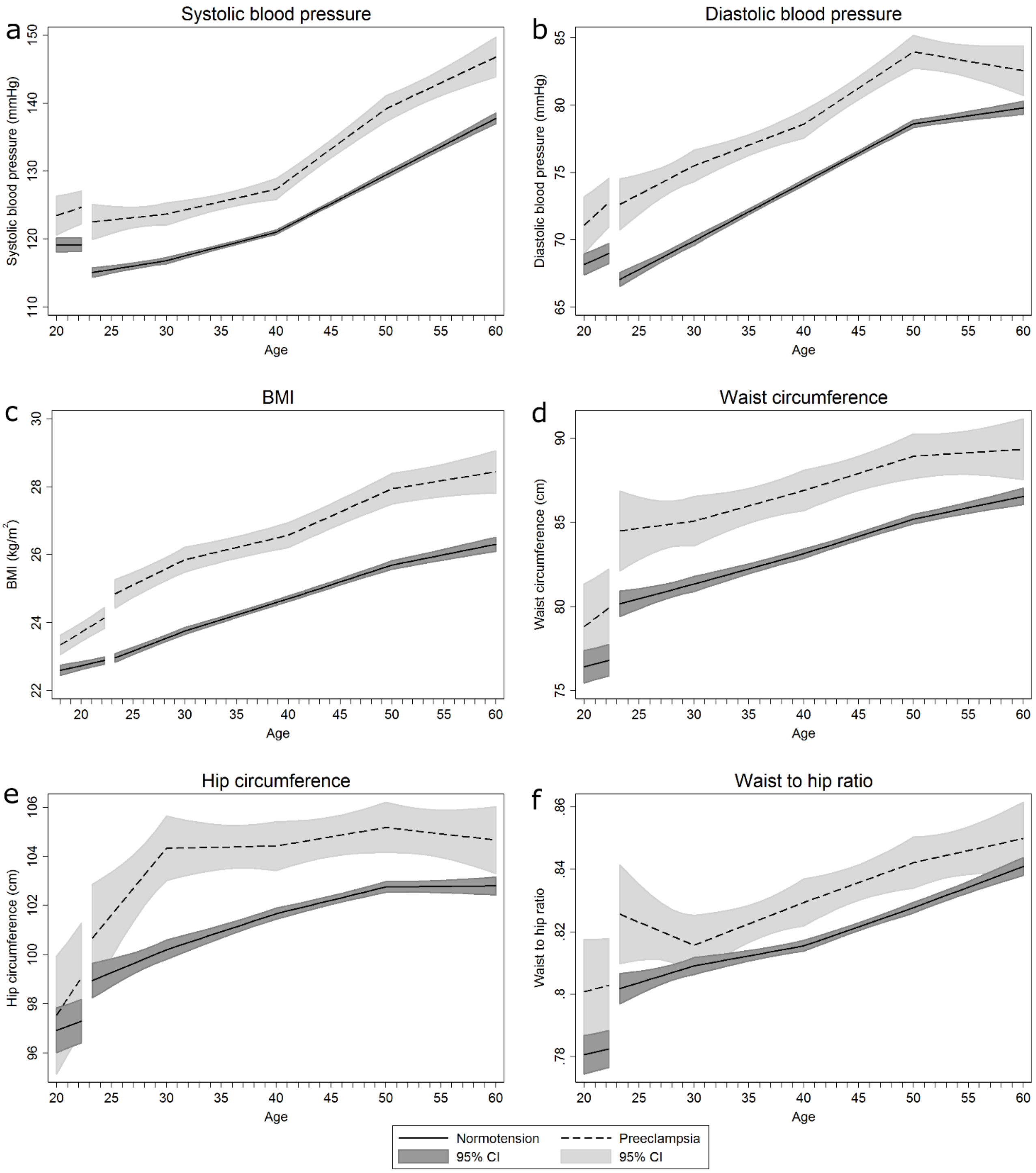

Estimates are adjusted for age at measurement, HUNT survey, highest obtained education level, age at first pregnancy and ever daily smoking. Covariates are fixed at their means with gaps in the graphs corresponding to the woman's first pregnancy, birth at age 23 and a three-month postpartum period.

Figure S6. Life course trajectories of mean non-fasting serum non-HDL (a) and HDL (b) cholesterol, triglycerides (c) and glucose (d), resting heart rate (e), and estimated glomerular filtration rate (f) for women with normotensive and preeclamptic first pregnancies who had two or more observations.

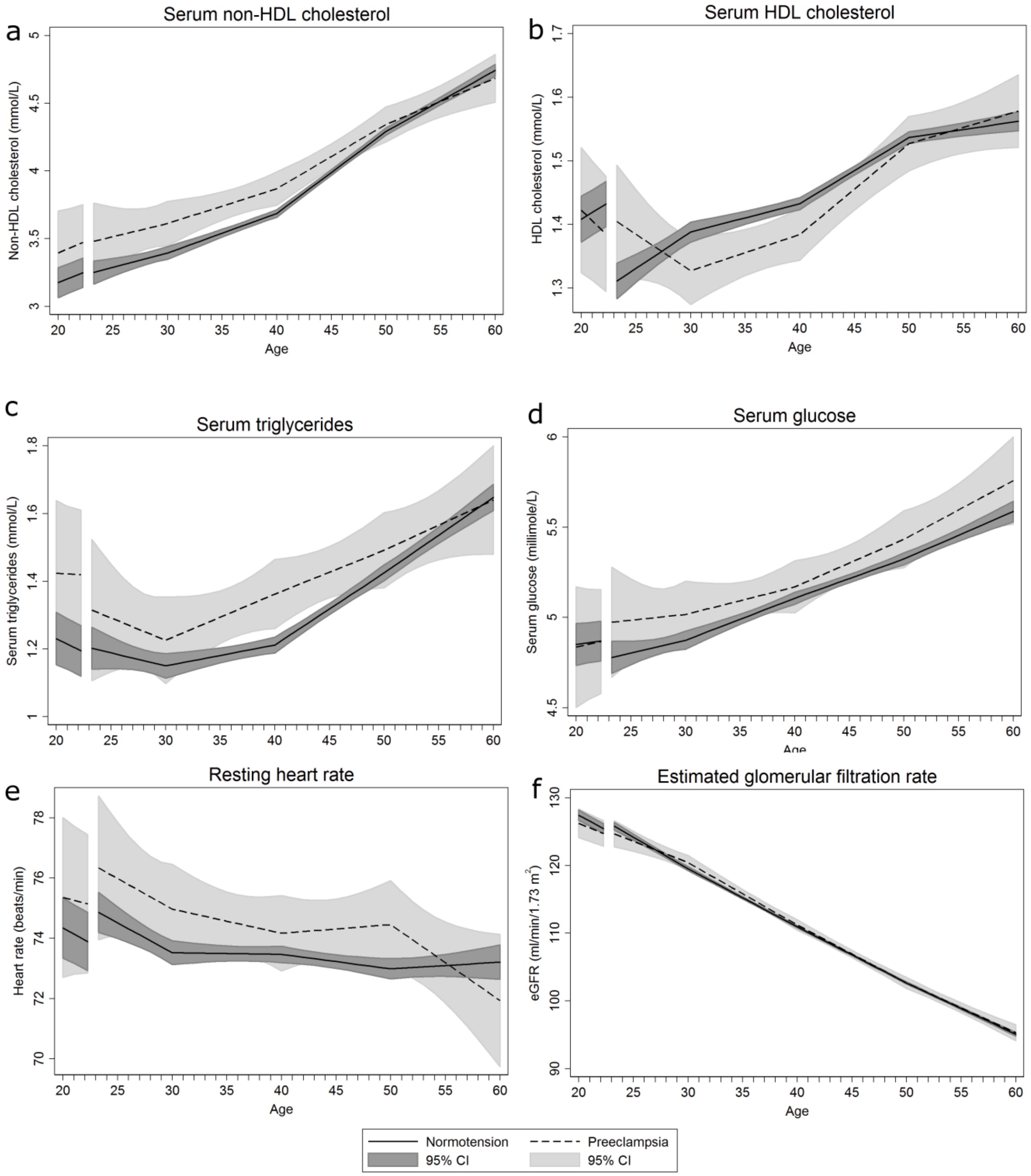

Estimates are adjusted for age at measurement, HUNT survey, highest obtained education level, age at first pregnancy and ever daily smoking. Covariates are fixed at their means with gaps in the graphs corresponding to the woman's first pregnancy, birth at age 23 and a three-month postpartum period.

Figure S7. Life course trajectories of mean systolic blood pressure (a), diastolic blood pressure (b), BMI (c), waist circumference (d), hip circumference (e) and waist to hip ratio (f) for women with normotensive first and second pregnancy, preeclampsia in one of their first two pregnancies and preeclampsia in both the first and second pregnancy.

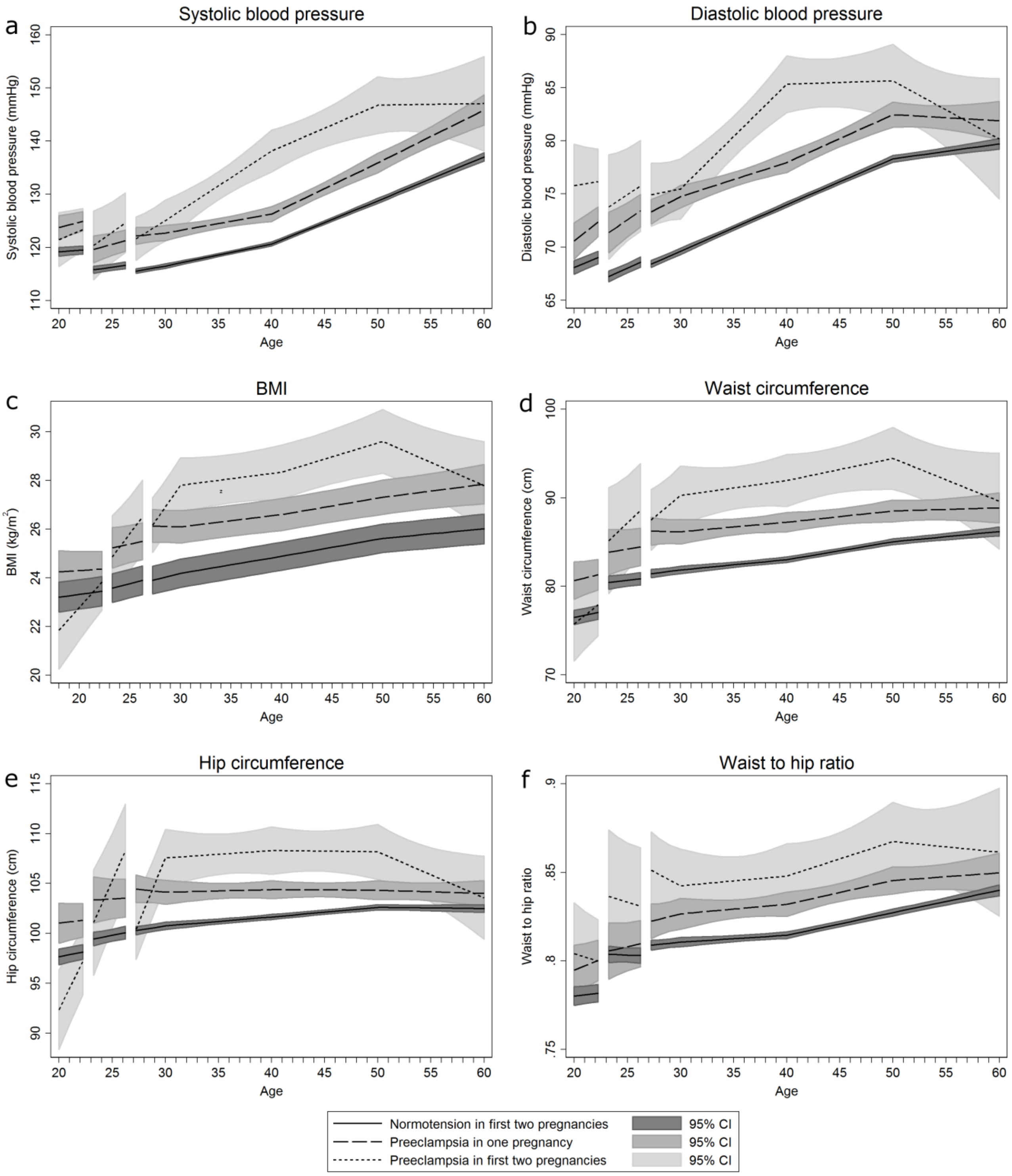

Estimates are adjusted for age at measurement, HUNT survey, time between first and second pregnancy, highest obtained education level, age at first pregnancy and ever daily smoking. Covariates are fixed at their means with gaps in the graphs corresponding to the woman's first and second pregnancy, birth at age 23 and 27 and three-month postpartum periods.

Figure S8. Life course trajectories of mean non-fasting serum non-HDL (a) and HDL (b) cholesterol, triglycerides (c) and glucose (d), resting heart rate (e) and serum CRP (f) for women with normotensive first and second pregnancy, preeclampsia in one of their first two pregnancies and preeclampsia in both the first and second pregnancy.

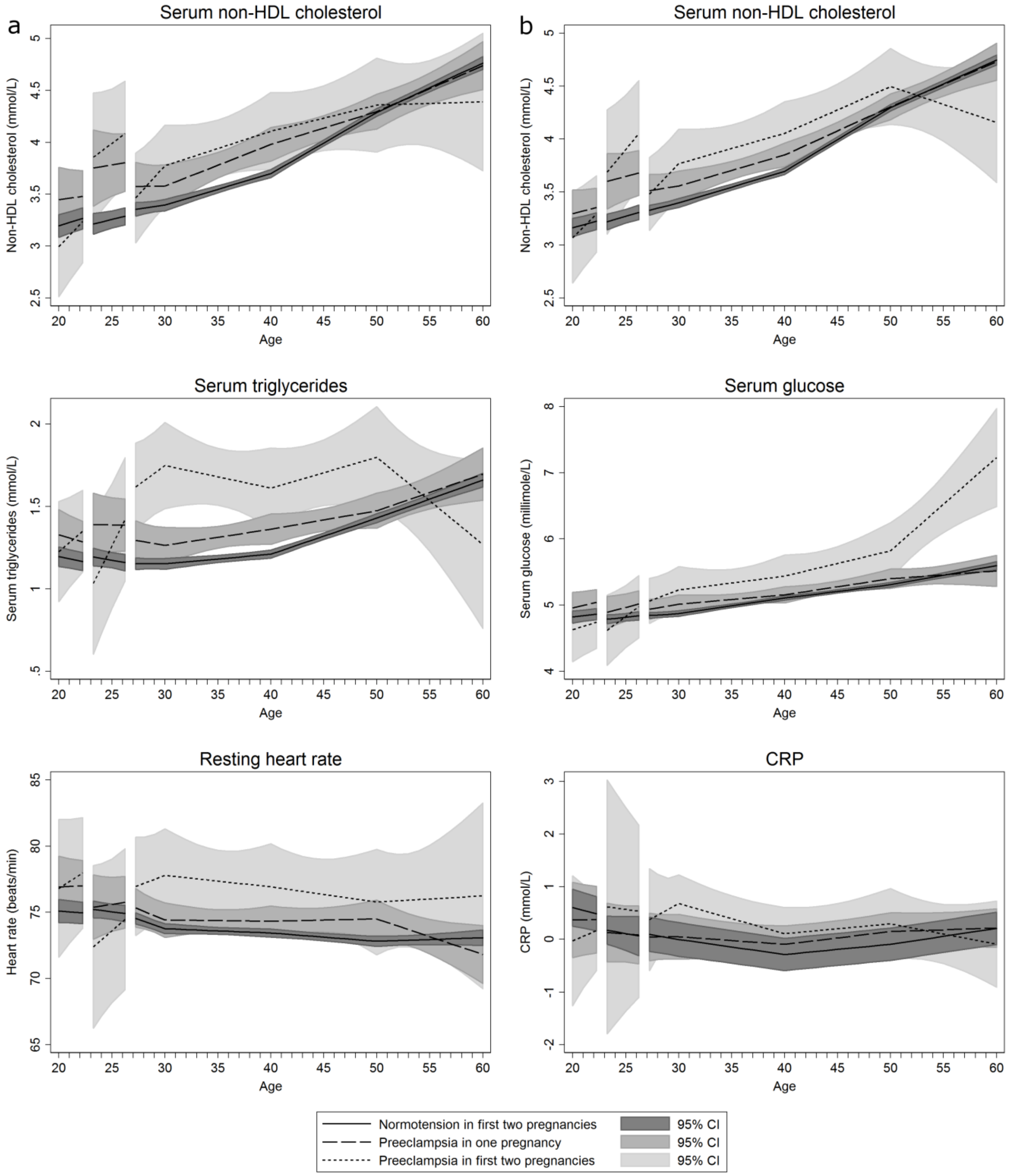

Estimates are adjusted for age at measurement, HUNT survey, time between first and second pregnancy, highest obtained education level, age at first pregnancy and ever daily smoking. Covariates are fixed at their means with gaps in the graphs corresponding to the woman's first and second pregnancy, birth at age 23 and 27 and three-month postpartum periods.

Figure S9. Life course trajectories of mean estimated glomerular filtration rate (eGFR) for women with normotensive first and second pregnancy, preeclampsia in one of their first two pregnancies and preeclampsia in both the first and second pregnancy.

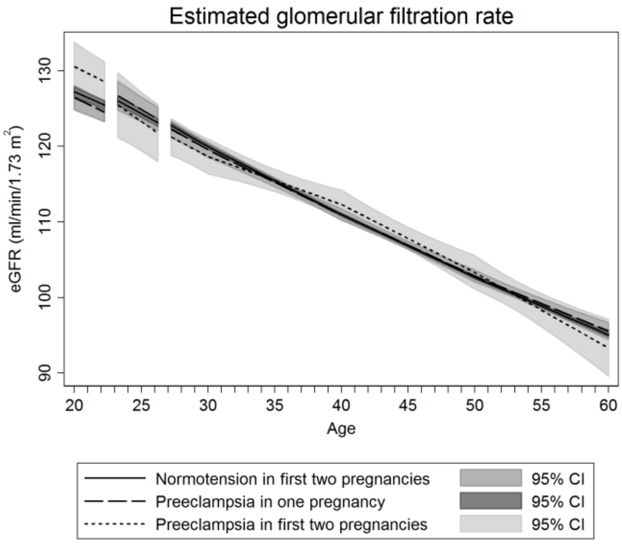

Estimates are adjusted for age at measurement, HUNT survey, time between first and second pregnancy, highest obtained education level, age at first pregnancy and ever daily smoking. Covariates are fixed at their means with gaps in the graphs corresponding to the woman's first and second pregnancy, birth at age 23 and 27 and three-month postpartum periods.

Figure S10. Population average predicted probabilities of hypertension (defined as current antihypertensive medication and/or blood pressure ≥140 mmHg systolic or ≥90 mmHg diastolic) (a) and obesity (defined as a BMI ≥30 kg/m^2) (b) by age in women with normotensive 1st and 2nd pregnancy, preeclampsia in one of their first two pregnancies and preeclampsia in both the 1st and 2nd pregnancy.

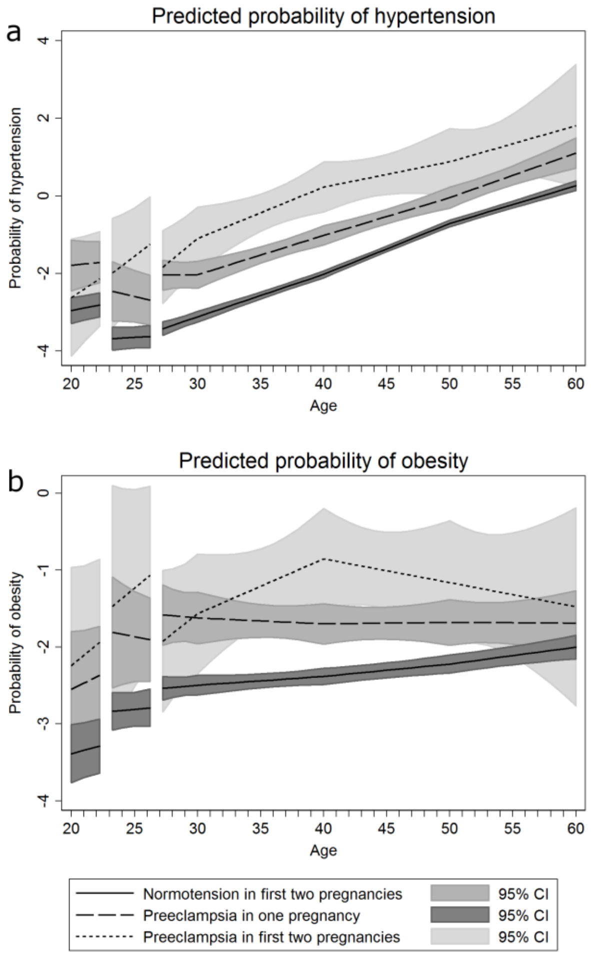

Estimates are adjusted for age at measurement, HUNT survey, time between 1st and 2nd pregnancy, highest obtained education level, age at first birth and ever daily smoking. Covariates are fixed at their means with gaps in the graphs corresponding to the woman's first pregnancy, birth at age 23 and a three-month postpartum period.
